# Optimization, purification and characterization of laccase from a new endophytic *Trichoderma harzianum* AUMC14897 isolated from *Opuntia ficus-indica* and its applications in dye decolorization and wastewater treatment

**DOI:** 10.1186/s12934-024-02530-x

**Published:** 2024-10-05

**Authors:** Maha M. Salem, Tarek M. Mohamed, Aya M. Shaban, Yehia A.-G. Mahmoud, Mohammed A. Eid, Nessma A. El-Zawawy

**Affiliations:** 1 Biochemistry Division, Chemistry Department, Faculty of Science, Tanta University, Tanta, 31527 Egypt; 2 Botany and Microbiology Department, Faculty of Science, Tanta University, Tanta, 31527 Egypt

**Keywords:** Endophytic fungi, Ovat-statistical laccase optimization, Purification, Characterization, Decolorization

## Abstract

**Background:**

Hazardous synthetic dye wastes have become a growing threat to the environment and public health. Fungal enzymes are eco-friendly, compatible and cost-effective approach for diversity of applications. Therefore, this study aimed to screen, optimize fermentation conditions, and characterize laccase from fungal endophyte with elucidating its ability to decolorize several wastewater dyes.

**Results:**

A new fungal endophyte capable of laccase-producing was firstly isolated from cladodes of *Opuntia ficus*-*indica* and identified as *T. harzianum* AUMC14897 using ITS-rRNA sequencing analysis. Furthermore, the response surface methodology (RSM) was utilized to optimize several fermentation parameters that increase laccase production. The isolated laccase was purified to 13.79-fold. GFC, SDS-PAGE revealed laccase molecular weight at 72 kDa and zymogram analysis elucidated a single band without any isozymes. The peak activity of the pure laccase was detected at 50 °C, pH 4.5, with thermal stability up to 50 °C and half life span for 4 h even after 24 h retained 30% of its activity. The K_m_ and V_max_ values were 0.1 mM, 22.22 µmol/min and activation energy (E_a_) equal to 5.71 kcal/mol. Furthermore, the purified laccase effectively decolorized various synthetic and real wastewater dyes.

**Conclusion:**

Subsequently, the new endophytic strain produces high laccase activity that possesses a unique characteristic, it could be an appealing candidate for both environmental and industrial applications.

**Supplementary Information:**

The online version contains supplementary material available at 10.1186/s12934-024-02530-x.

## Background

The globe is undergoing tremendous technological improvement, which has resulted in increased pollution, notably in water, due to various hazardous chemicals such as pesticides, detergents, and synthetic dyes [1]. Dyes that effluent from the industrial factories poses significant environmental challenges due to the recalcitrant nature of synthetic dyes and their potential toxicity to aquatic ecosystems. Also, dyes have an extremely complicated structure that makes them hazardous and perhaps carcinogenic [2]. It has been discovered that the current physical and/or chemical processes, such as adsorption, precipitation, and chemical degradation, are costly, harmful to the environment, and ineffective for decolorizing dyes. Therefore, there is a pressing need for an economical and environmentally friendly method of treating water [3].

Recently intense research has been concentrated on biodegradable enzymes instead of the physical/chemical water treatment methods. Laccase (benzenediol: oxygen oxidoreductase, EC 1.10.3.2) is one of the biodegradable enzymes. It has been claimed that the multicopper enzyme laccase can be used to treat industrial and wastewater effluent by catalyzing the reduction of O_2_ to H_2_O with the existence of substrates [4]. Numerous benefits of laccase include its broad substrate specificity, high catalytic efficiency, and tolerance to a range of chemical and physical conditions, making it a promising candidate for dye decolorization [5]. However, barriers to large-scale laccase manufacturing include expensive production costs and the lack of an effective expression method. Therefore, finding novel laccase sources and developing affordable techniques for their quick extraction are crucial.

Production of microbial enzymes is efficient because it is rapid, inexpensive, scalable, and open to genetic alterations [6]. Among microbial sources, fungi are considered an intriguing source for industrial enzymes [7, 8]. Fungal enzymes are known for their exceptional stability, high production potency, and ease of purifying and separation processes [9]. Moreover, endophytic fungi have been investigated as potential sources of hydrolytic enzymes [10, 11]. Endophytic fungi are common in plants, living in plant tissues without causing any visible symptoms in their hosts [12]. The presence of these fungi in plant tissues under extreme conditions may explain their ability to create compounds with useful industrial, agricultural, and medical uses [13]. Only endophytic *Myrothecium verrucaria* have been reported to generate laccase and there are very little reports about laccase production from endophytic fungi [14]. Endophytic fungal strains may thus be a rich biological source that should be examined and investigated for laccase enzyme synthesis. Therefore, the current study focused on the identification of endophytic fungi from *Opuntia ficus-indica* as laccase producer for the first time, followed by optimization, purification, and biochemical characterization of laccase to assess its wastewater dyes decolorization performance.

## Materials and methods

### Chemicals

ABTS (2.2-azino bis (3 ethyl benzothiazoline) 6- sulfonic acid diammonium salt, 98% (HPLC) (cat# A1888) was obtained from (Sigma-Aldrich Pvt Ltd. USA), potato dextrose agar (PDA) (cat#70139), peptone (cat#P6838), glycine (cat# G8898), malt extract (cat# 1.05391) and ammonium sulphate (cat# A4418) were purchased from (Merck, USA). Light Green SF Yellowish 70% (CAS #5141-20-8), Cresol red 95% (CAS# 1733-12-6), Malachite Green 90% (CAS#569-64-2), Aniline Blue 75% (CAS# 66687-07-8), Tartrazine 85% (CAS#1934-21-0), Methylene Blue 70% (CAS# 122965-43-9), were obtained from Sigma-Aldrich and Fast turquoise blue 95% (CAS# 1330-38-7) was purchased from Qingdao Sanhuan Colorchem Co., Ltd. All other chemicals used were of high grade.

### Source of fungi and culture maintenance

Three different strains of endophytic fungi encoded as (F-1, F-2, and F-3) were previously obtained from cladodes of *Opuntia ficus-indica* (L.) Mill. (Cactaceae) [15]. Strains were cultivated on petri plates using potato dextrose agar (PDA; Ifco Laboratories, Detroit, MI, USA) with chloramphenicol (300 µg/mL) to prevent bacterial growth. After 5 days of incubation at 30 °C, the plates were stored at 4 °C for future use.

### Screening of laccase producing endophytic fungus

The selected endophytic fungal strains were inoculated to PDA petri dishes supplemented with 0.01% guaiacol (w/v) as lacasse indicator. After incubation at 30 °C, all plates were examined daily until the formation of reddish-brown halo zone was detected. The ratio between the size of the colored halo and the size of the colony was recorded as an indication to screen positive laccase strain for further studies [9].

### Identification of laccase producing endophytic fungus

Positive laccase endophytic fungus utilized in this work was identified based on the traditional morphological characteristics. Aerial mycelium and density were recorded as features of the colonies. The micro-morphological characteristics were analyzed using a light microscope (Olympus CX51, Japan) following the method previously reported [16].

The DNA/RNA extraction kit, supplied by the Korean company Intron Biotechnology, was used to extract DNA from cultures delivered to the Molecular Biology Research Unit at Assiut University to verify the identification of the laccase-producing endophytic fungi. For PCR and rRNA gene sequencing, fungus DNA samples were delivered to Sol Gent Company in Daejeon, South Korea. The reaction mixture contained ITS1 (forward) and ITS4 (reverse) primers, which were used for PCR. Primers are composed of the following: "ITS1 (5′-TCC GTA GGT GAA CCT GCG G-3′), and ITS4 (5′- TCC TCC GCT TAT TGA TAT GC -3′)" [17]. Using BLAST, the obtained sequences were compared to the sequences deposited in the NCBI database to ascertain similarity with the closest related species. The neighbor-joining phylogenetic tree for the ITS rRNA gene was made using MEGAX program [18].

### Optimization of cultural growth conditions for positive laccase endophytic fungus

For fermentation, a modified basic nutrient Potato Dox medium was used. The composition of the medium used in the study conducted by Abd El-Rahim et al. [19], consisted of the following components per liter: 200 g of potato, 15 g of glucose, 2 g of peptone, 3 g of yeast extract, 3 g of KH_2_PO_4_, and 1.5 g of MgSO_4_.7H_2_O. Then, one variable at a time method (OVAT) was principally used to screen and optimize different factors related to laccase synthesis as described below.

### Effect of incubation period, temperature, and pH

Each fermentation broth (100 mL) with three mycelia discs of selected strain was incubated for 10 days to measure the laccase production daily. Then, each culture flask (100 mL) with three mycelia discs of selected strain was incubated for 6 days at different temperature (20–40 °C) and pH (3.0–9.0).

### Effect of different carbon and nitrogen sources

Liquid fermentation broth (pH = 6) was modified by changing the concentration and nature of nutritional sources. Carbon and nitrogen sources weighing 15 g/L (glucose, fructose, sucrose, and maltose) and 2 g/L (peptone, ammonium sulphate, ammonium chloride, sodium nitrate and glycine) were investigated individually to obtain the best carbon or nitrogen source suitable for high laccase production. Moreover, different concentrations of the best carbon (5–25 g/L) and nitrogen (0.5–10 g/L) sources were tested. The flasks were incubated for 6 days at 28 °C.

### Response surface optimization

To achieve efficient laccase production, the positive laccase endophytic fungus was optimized using a central composite design (CCD) with response surface methodology (RSM). Fermentation temperature (≦C), pH, and time (T) were selected as the three independent variables to investigate their effects on laccase production. Twenty experimental sets in total were constructed to fit a complete quadratic equation model. Equation (1) was given as below:

1 $${\text{Y}}\, = \,\beta_{0} \, - \,\sum \beta_{{\text{j}}} {\text{X}}_{{\text{j}}} \, + \,\sum \beta_{{\text{j j}}} {\text{X}}_{{\text{j}}}^{{2}} \, + \,\sum \sum \, \beta_{{{\text{ij}}}} {\text{X}}_{{\text{i}}} {\text{X}}_{{\text{j}}}$$

where, Y represented dependent variable; 0, j, jj, and ij were the regression coefficients for square, interaction, linearity, and intercept, respectively; the independent coded variables were Xi and Xj. Design-Expert 13.0 was used to analyze the real data and the anticipated responses as shown in Table1. Three-dimensional surface plots and analysis of variance (ANOVA) were utilized to determine the ideal fermentation conditions for the chosen isolate's synthesis of laccase.

**Table 1:** Experimental variables for central composite design at different levels

Factors	Variables	Units	Experimental values		
			Low level (−1)	Intermediate level (0)	High level (+ 1)
A	Time	Days	2	5	6
B	Temperature	◦C	28	30	32
C	pH	–	5	6	7

### Assay of laccase activity

The laccase activity was measured according to [20] depending on the ABTS's oxidation. The rate of ABTS oxidation was measured at 436 nm. Briefly, the reaction mixture consists of 100 µL sodium acetate buffer (50 mM, pH 5.4), 500 µL ABTS (50 mM) and 400 µL culture filtrate (enzyme source) and was incubated at 37 ºC for five minutes. The amount of enzyme required to oxidize 1 μmol of ABTS per minute is known as laccase activity. The Bradford technique was used to estimate the protein content using bovine serum albumin (BSA) as a standard [21].

### Purification of laccase enzyme

Batch inoculated culture broth was incubated at optimum production conditions, then, cell free supernatant was extracted using centrifugation at 4 °C for 15 min at 6000 rpm, which considered as crude laccase. The first step of laccase purification was using 80% ammonium sulfate fractionation. The precipitate was recovered after spinning at 6000 rpm for 15 min at 4 °C, then it was dissolved in the smallest quantity of 50 mM acetate buffer (pH 5.4). Following, Sephacryl 300 HR gel filtration chromatography (GFC) was used and mounted vertically on a suitable ring stand. The column beds were washed several times with distilled water, then with 2–3 bed volumes of 50 mM acetate buffer, pH 5.4 to pack the bed and to equilibrate the column with buffer. The laccase enzyme after ammonium sulphate precipitation was loaded on the gel. The amount of buffer used for elution was two volumes of resin beds. A flow rate of 3 mL/5 min was used to elute the proteins. [22]. At 280 nm, the total amount of protein in each fraction was measured.

### Determination of molecular weight using gel filtration chromatography

The calibration curve for molecular weight determination by gel filtration chromatography can be prepared by individually applying and eluting five suitable standard proteins over the column using "Gel Filtration Markers Kit for Protein Molecular Weights 12,000–200,000 Da" (Sigma-Aldrich (cat#MWGF200). The logarithm of molecular weight of each standard proteins were plotted versus *V* _e_ /*V* _0_. Therefore, the isolated laccase molecular weight could be predicted from the calibration curve under the same conditions [23, 24].**‏** The molecular weight protein markers used in kit were cytochrome-C, horse heart (12.4 kDa), Carbonic anhydrase, Bovine erythrocyte (29 kDa), Albumin, Bovine serum (66 kDa), Alcohol dehydrogenase, yeast (150 kDa) and ß-Amylase, sweet potato (200 kDa).

### Molecular mass

#### Polyacrylamide gel electrophoresis using sodium dodecyl sulfate (SDS-PAGE)

For SDS-PAGE gel preparation, an equal amount of different laccase purification steps (crude, 80% ammonium sulfate pellet, and Sephacryl column eluting factions) were mixed with a 3 × loading buffer (3.0 mL of distilled water, 1.2 mL of 1 M Tris–HCl at pH 6.8, 2.4 mL of glycerol, 0.48 g of SDS, 60 μL of 10% bromophenol blue solution, and 1.5 μL of β-mercaptoethanol). These mixtures were subsequently incubated at a temperature of 100 °C for 5 min and were then directly loaded onto SDS-PAGE mini gel (6 cm × 9 cm). The electrophoresis process was run at 100 V using 1 × running buffer (14.4 g of glycine and 3 g of Tris-base dissolved in 1L of distilled water). The electrophoresis process was considered complete when the bromophenol blue dye had migrated to the bottom of the gel. After running, the power supply was switched off and the gel plates were taken out. After removing the gel and soaking it in the staining solution for a whole night, it was shaken and immersed in the destaining solution until the bands were clearly visible. By comparing the observed protein bands with the molecular weight protein marker ladder, the estimated molecular weight of the bands was ascertained [25].

#### Native PAGE zymographic analysis

A zymogram was performed to analyze laccase isozymes in crude extract, ammonium sulfate precipitated fraction, and gel filtration chromatography purified protein samples. 20 μg of each sample was loaded onto a 7 % non-denaturing polyacrylamide gel and separated by electrophoresis at 60 volts for 48 h. After electrophoresis, the gel was incubated in a staining solution containing 50 mM acetate buffer, pH 4.5 and 1 mM ABTS a substrate for laccase, at 37 °C for 5 minutes. This allowed the laccase enzyme present in the samples to oxidize ABTS, forming a green colored product. The zymogram banding patterns of lane 1 (crude extract), lane 2 (ammonium sulfate fraction), and lane 3 (gel filtration fraction) were compared to assess relative laccase isozymes and effectiveness of the purification steps [26].

### Biochemical characterization of purified laccase activity

#### Effect of temperature

Using conventional assay procedures, the reaction mixture was incubated at different temperatures ranging from 20 to 90 °C to determine the ideal reaction temperature for pure laccase**.** The residual enzymatic activities were then ascertained using the method previously mentioned [20, 27]**.‏**

#### Evaluation of pure laccase half life span

The thermo-tolerant nature of the pure laccase was evaluated to ensure its applicability in harsh industrial conditions by measuring the half-life span. The half-life span was carried out at different temperature values (40–80 °C) with variable time intervals (0.5–4 h and 24 h). The activity of laccase was recorded under standard assay conditions as previously mentioned [28, 29]**.**

#### Effect of different pH values and enzyme concentrations

Different pH values were studied for isolated pure laccase with ABTS as a substrate in the range of 3.5–10.6 under standard assay conditions. The pH of pure laccase was adjusted using various buffers such as acetate buffer (50 mM; pH 3.5–5.8), phosphate buffer (50 mM; pH 6.2–8) and carbonate/ bicarbonate buffer (50 mM; pH 9.2–10.6) [30]**.** Furthermore, different concentrations of the pure laccase ranging from (0.045–61 U/mg) were added to the reaction mixture under standard assay conditions [31]**.**

### Evaluation of Kinetic parameters

#### Determination of K_m_ and V_max_

By evaluating the laccase activity at various ABTS concentrations (0.02–0.1 mM), the kinetic features of the isolated pure laccase, including its maximum velocity (Vmax) and Michaelis constant (Km), were revealed. Lineweaver and Burk's double-reciprocal plot graphical approach was utilized to calculate the Km and Vmax values [32, 33].

#### Determination of the activation energy (E_a_)

Using ABTS as a substrate and tracking the maximum initial rate (V) at different temperatures (T), the activation energy (Ea) of the purified laccase was determined by calculating the slope of the linear Arrhenius plot of log V against 1/T (K^−1^), where Ea =−slope × 2.3R and R (gas constant) = 1.987 × 10^–3^ kcal/mol [34].

#### Effect of different metal ions and chelating EDTA

Effects of several mono, di, trivalent cations (K⁺, Mg^2+^, Hg^2^⁺, Ca^2^⁺, Cu^2^⁺, and Al^3^⁺) and ethylene di amine tetra acetic acid (EDTA) at concentrations (1 and 2 mM each) on the activity of purified laccase were evaluated. The relative activity (%) was detected by laccase pre-incubation with the additives mentioned above and compared with respect to no-additive control under ideal assay conditions as previously mentioned [28].

### Wastewater treatment

#### Synthetic dyes decolorization

Decolorization was conducted using purified laccase on seven different dye types based on their chemical structure. Table S1 showed different kind of dyes with their structures and molecular weight as triarylmethane (cresol red, light green SF yellowish and malachite green), thiazine (methylene blue), triphenyl methane (aniline blue), azo dye (tartarazine) and a phthalocynaine dye (fast turquoise blue) with concentration (50 mg/L). Each kind of dye was incubated with purified laccase at different time intervals (30 min, 1 h, and 24 h) using 50 mM acetate buffer (pH 4.5) at 50 ºC. The disappearance of the color was monitored at λ_max_ for each dye respectively. The percentage of decolorization was determined as the Eq. (2) mentioned.

2 $$Decolorization \, percentage \, \left( \% \right)\, = \frac{{\left( {Ac - At} \right)}}{Ac}\, \times \,100$$

where A_t_ is the laccase test sample's absorbance and A_c_ is the control's absorbance [35].

#### Environmental real wastewater dye decolorization samples

Three different real industrial wastewater samples were collected from various dyeing and weaving factories in EL-Mahala, EL-Gharbia, Egypt, to study the ability of isolated purified laccase derived from *T. harzianum* AUMC14897 for their decolorization. These dyes are Vat dye as Novatic green XBN, azo dye as Red 4BL and Reactive T. Blue G 266% dye (Table S1). The *T. harzianum* AUMC14897 laccase was incubated with these different wastewater samples at different time intervals (30 min, 1 h, and 24 h) using 50 mM acetate buffer (pH 4.5) at 50 ºC. The previously given equation was utilized to compute the dye decolorization capacity of pure laccase [36].

### Statistical analysis

The investigations were conducted in three separate instances, and the mean ± SD of the outcomes was reported, (n = 3). A one-way ANOVA was used to determine significant differences. *p* < 0.05 indicated significant differences.

## Results and discussion

Advanced industrialization has led to a surge in the continuous discharge of hazardous effluents to the environment; particularly from industrial factories generating vast volumes of recalcitrant dye-containing wastewater. In response, our study explored the bioremediation strategies employing microbial laccase for its eco-friendly and cost-effective nature. Laccase is one of the enzymes that involved in various aspects of remediation such as waste detoxification and dye decolorization [37]. Therefore, our study focused on endophytic-derived laccase and its potential application in wastewater treatment via dye decolorization.

### Screening and identification of the most potent laccase-producing endophytic fungus

**Fig. 1:**
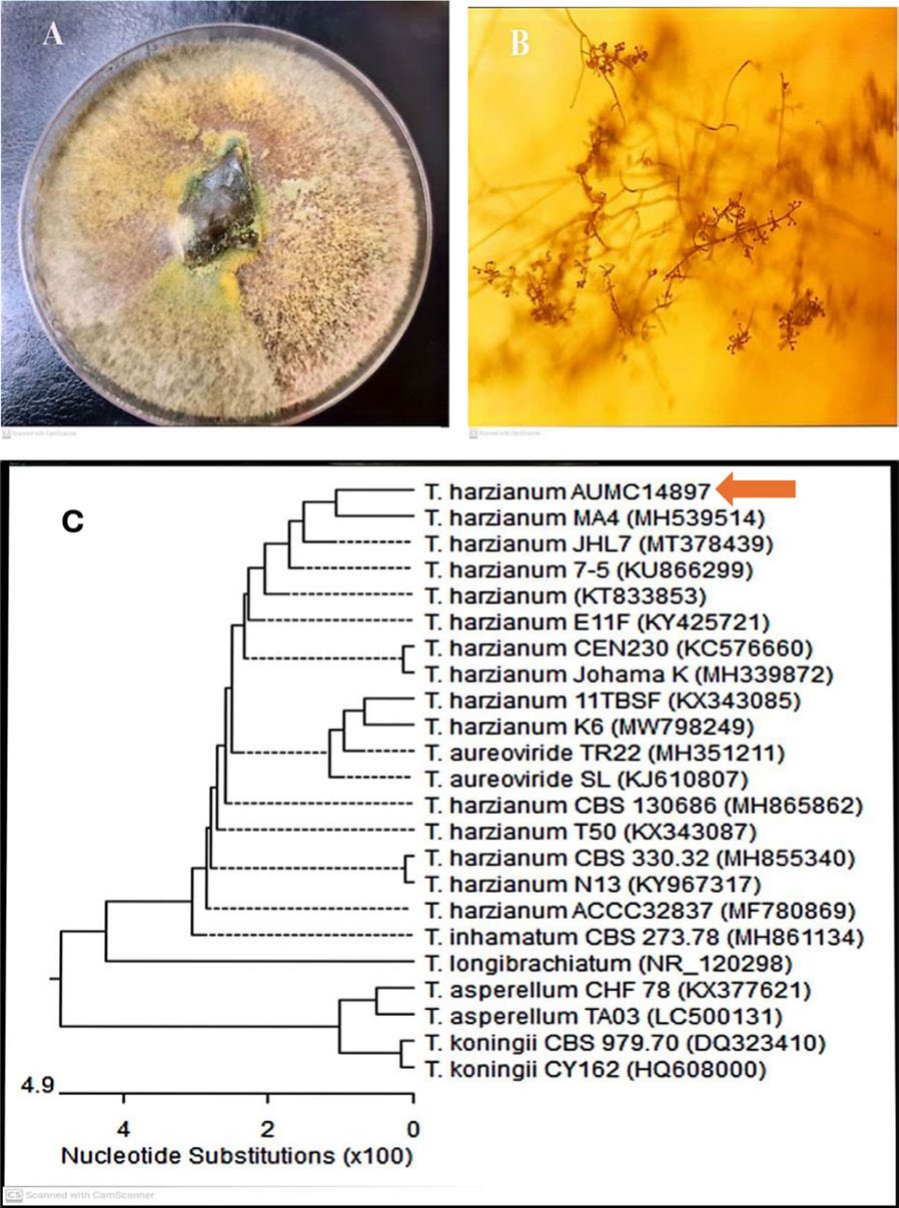
Morphological and molecular identification of lacasse-producing fungal endophyte.** A** and **B** represented colonial morphology and micrographic characteristics (× 10), respectively. **C** A phylogenetic tree established from the ITS sequences of the 18S rDNA of lacasse- producing fungal endophyte (*Trichoderma harzianum* AUMC14897; GenBank accession number MZ025966)

A reliable marker and observable screening step for laccase activity is guaiacol. The halo zone with reddish-brown color was regarded as a positive result of guaiacol oxidation process via laccase enzyme [38]. In our study, the three selected endophytic fungal strains which encoded as F-1, F-2, F-3 as in (Fig S.1), were preliminarily screened to produce laccase enzyme on PDA plates contained guaiacol indicator. After 5 days at 30 °C, only the F-1 strain showed a distinct reddish brown colored zone surrounding the colony as shown in the supplementary file (Fig S.1B). On the other hand, F-2 and F-3 strains had negative potential for laccase production (Fig S.1E). Similarly, Sun, et al. [14] detected laccase enzyme from *Myrothecium verrucaria* MD-R-16 using guaiacol as indicator. Our results revealed that F-1 strain was chosen for morphological and molecular identification, as well as further laccase extraction and characterization. Colonies of F-1 strain showed whitish mycelia which changed to green upon sporulation (Fig. 1A). In addition, conidia were globose to sub globose, initially white, and subsequently pale green with flask shaped phialides (Fig. 1B). Based on morphological observations, this strain was identified as *Trichoderma* sp. which was taxonomically identified by amplifying the ITS-rDNA genes using PCR. ITS is often used to investigate relationships across different fungus, especially among closely related species [39]. Based on phylogenetic analysis (Fig. 1C), F-1 strain was identified as *Trichoderma harzianum* AUMC14897 which has been deposited in GenBank under accession number (MZ025966) and showed 100% identity to *T. harzianum* strain MA4 (MH539514).

### Optimization of laccase production from *T. harzianum* using OVAT method

The production of laccase is significantly influenced by common parameters, including pH, temperature, incubation period, and carbon and nitrogen supplies [40]. In our work, we mostly investigated these issues with a single variable approach.

### Effect of different incubation time, temperature, and pH

**Fig. 2:**
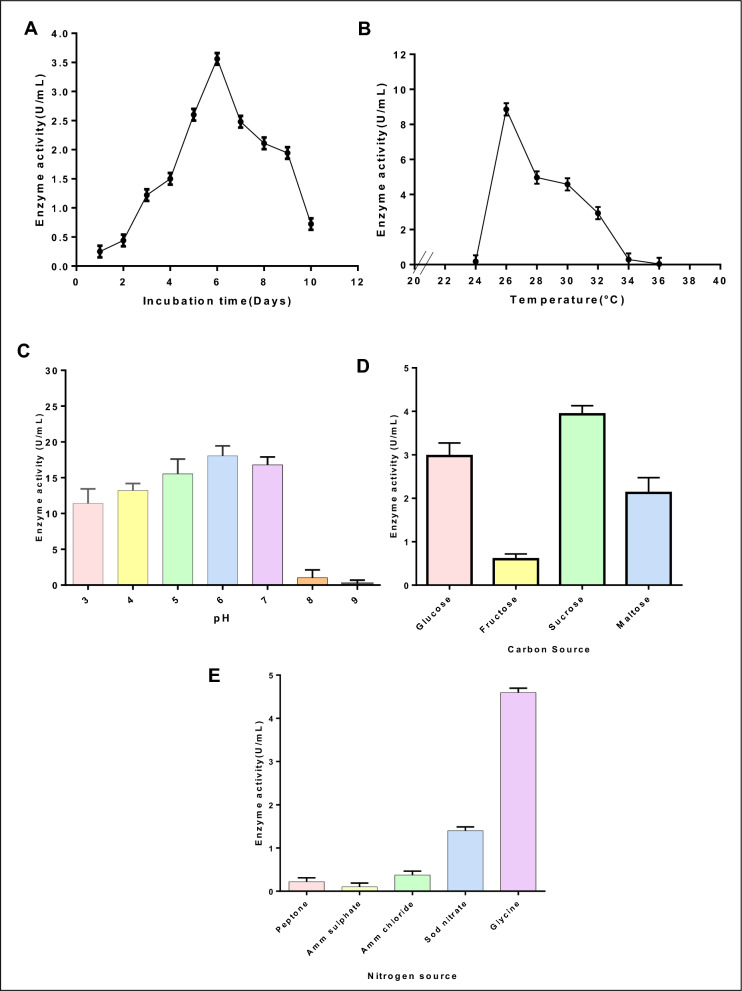
Effect of different factors on laccase production by *T. harzianium* AUMC14897 **A** Different carbon sources concentrations **B** Different nitrogen sources for laccase production from *T.harzianum*. **C** Different incubation times (days). **D** Different range of temperature*.* **E** Different pH values for laccase production from* T.harzianum*

To increase laccase production, several factors were examined, including temperature, time, and pH, which are important for microbial cell metabolism. Figure 2A showed highest laccase production by *T. harzianum* AUMC14897 on the sixth day giving 3.56 U/mL. On the sixth day of incubation, the biomass of fungal mycelium covered the liquid medium and released the highest amount of laccase, and then the activity decreased to 0.722 U/mL after 10 days. Similar characteristics were noted in *Ganoderma leucocontextum* broth that was entirely covered by mycelium biomass on day six and seven giving high laccase activity (27 U/L) [31]. Also, on the fifth day, the endophytic fungus *Irpex lacteus* reached its maximal activity of laccase (38.25 U/L) [41]. The laccase production initially increased by increasing fermentation time and temperature, and then decreased with the longer fermentation time and higher fermentation temperature. This may be attributed to the fact that the nutrients in the medium could be gradually used up, which could affect the fungi physiology that led to the inactivation of the secretary machinery of enzymes [42]. In contrast, laccase produced from *Marasimus palmivorus* showed high production after the third day of incubation with activity (0.922 U/mL**) **[43].

In our study, at 28 °C, laccase peaked high activity (8.861 U/mL), further laccase synthesis decreased when the temperature increased to 36 °C (0.041 U/mL) (Fig. 2B). Similar temperature preferences were observed for laccase from *Penicillium chrysogenum* which had maximum activity (7.9 U/mL) at 32 °C as mentioned by Senthivelan et al. [44].

pH value varies based on the origin of the laccase enzyme. High pH levels cause the substrate's redox potential to drop, and laccase activity is inhibited as a hydroxide anion binds to the type 2/type 3 coppers [45]. Results in Fig. 2C revealed an increase in laccase activity when the pH was raised from 3.0 to 6.0. Following that, laccase activity decreased. The optimal parameters yielded the maximum laccase production at the highest degree of laccase activity (17.4 U/mL) was obtained at pH 6.0 and complete inhibition at pH 9 (0.194 U/mL). This aligned with findings from other studies on *Marasimus palmivorus* and *Schizophyllum* commune laccases [43].

### Effect of various carbon and nitrogen sources with different concentrations

Laccase activity is influenced by the type and quantity of carbon sources. Due to the catabolites' repression, an abundance of sugar decreased the enzyme production [46, 47]. Laccase production reached its maximum extent of 3.93 U/mL, as seen in Fig. 2D when sucrose was used as the carbon source at 10 g/L concentration**,** while complete inhibition of laccase was observed with fructose, yielding an activity of 0.597 U/mL**.**

Similar observations were made by Thakkar & Bhatt [48] for *Aternaria alternata* laccase using glucose in which high laccase production was detected using glucose (1%) as carbon source with activity (69.08 U/mL). Additionally, Kumar & Prasher [49] reported varying laccase activities from *Diaporthe phaseolorum* with sucrose and fructose as carbon sources. In contrary to our findings, starch as carbon source with concentration 2% had high laccase activity (0.18 U/mL) produced by *Ganoderma sp.* as mentioned by Sivakumar et al. [50].

Laccase generation in wood-rotting fungi is influenced by the type and concentration of nitrogen sources [51]. Comparing different sources of nitrogen, specifically organic sources are more efficient [46]. Similar to our results, we revealed that glycine enhanced laccase production, reaching an activity 4.597 µmol/min/mL, while the inclusion of ammonium sulphate caused the laccase activity to drop to 0.1 U/mL (Fig. 2E). Contrastingly, laccase activity from *basidiomycetous* showed high activity with ammonium sulphate [50]. Moreover, sodium nitrate showed the lowest activity of laccase from *A. alternate* [47]. Furthermore, varying concentrations of the optimal carbon (sucrose) and nitrogen (glycine) sources, showed maximum laccase production in the presence of 10 g/L for both, resulting in 15.277 U/mL as shown in the supplementary file (Fig. S2A) and 16.5 U/mL as shown in the supplementary file (Fig. S2B), respectively. After ovat optimization, we found the initial pH, fermentation temperature, and time were three significant factors for laccase production. So, we use these parameters for central composite design of lacasse production to determine the optimal levels of these selected parameters.

### Optimization of laccase activity by RSM

According to Nyanhongo et al., [51], laccase synthesis by other microorganisms has been demonstrated to be impacted by temperature, pH, and duration time, all of which are significant factors in fungal fermentation. In our study we found that these factors also affected the laccase production significantly. The ideal quantity of each of the three given parameters was thus determined using CCD. For laccase activity, a second order polynomial equation was created based on the experimental data from CCD (Table 2), as indicated by the equation (3) that follows (regarding the real factors).

3 $${\text{R}}\, = \,{18}.{2752}\, + \,{3}.{367}\,*\,{\text{Time}}\, + \, - {2}.0{41}\,*\,{\text{Temperature}}\, + \,0.{845}\,*\,{\text{pH}}\, + \, - {6}.{76125}\,*\,{\text{Time}}^{{2}} \, + \, - {3}.{81125}\,*\,{\text{pH}}^{{2}}$$

**Table 2:** Central composite design results for laccase synthesis

Run No	Experimental parameters			Lacasse activity (U/mL)	
	A (Time, d)	B (Temperature, ◦C)	(pH)	Actual value	Predicted value
1	1	1	−1	8.03	8.18
2	−1	0	0	1.82	8.15
3	0	0	0	22.54	18.28
4	0	0	0	22.14	18.28
5	−1	−1	−1	5.63	5.53
6	1	0	0	9.55	14.88
7	−1	1	−1	4.01	1.45
8	1	−1	−1	15.95	12.27
9	0	0	0	22.21	18.28
10	0	0	0	22.38	18.28
11	1	1	1	10.12	9.87
12	0	0	−1	7.43	13.62
13	0	0	0	21.88	18.28
14	0	−1	0	15.68	20.32
15	−1	−1	1	6.44	7.22
16	0	1	0	9.05	16.23
17	0	0	0	21.98	18.28
18	1	−1	1	15.51	13.96
19	0	0	1	9.84	15.31
20	−1	1	1	7.59	3.14

Table 3 displayed the quadratic model's ANOVA results, which revealed a strong correlation between the response's experimental and anticipated values. The model's significant level (p<0.0001) was found by statistical analysis, suggesting that variation could potentially predict the model [39]**.**

**Table 3:** Laccase production using an ANOVA of the response surface quadratic model

Source	Sum of squares	df	Mean square	F value	P-value Prob > F	
Model	617.98	5	123.60	5.02	0.0077	Significant
A-time	113.37	1	113.37	4.60	0.0500	
B-temperature	41.66	1	41.66	1.69	0.2145	
C-Ph	7.14	1	7.14	0.2897	0.5988	
A^2^	146.29	1	146.29	5.94	0.0288	
C^2^	46.48	1	46.48	1.89	0.1912	
Residual	345.00	14	24.64			
Lack of Fit	344.70	9	38.30	634.77	< 0.0001	Significant
Pure Error	0.3017	5	0.0603			
Cor Total	962.98	19	R^2^ = 0.6417			

**Fig. 3:**
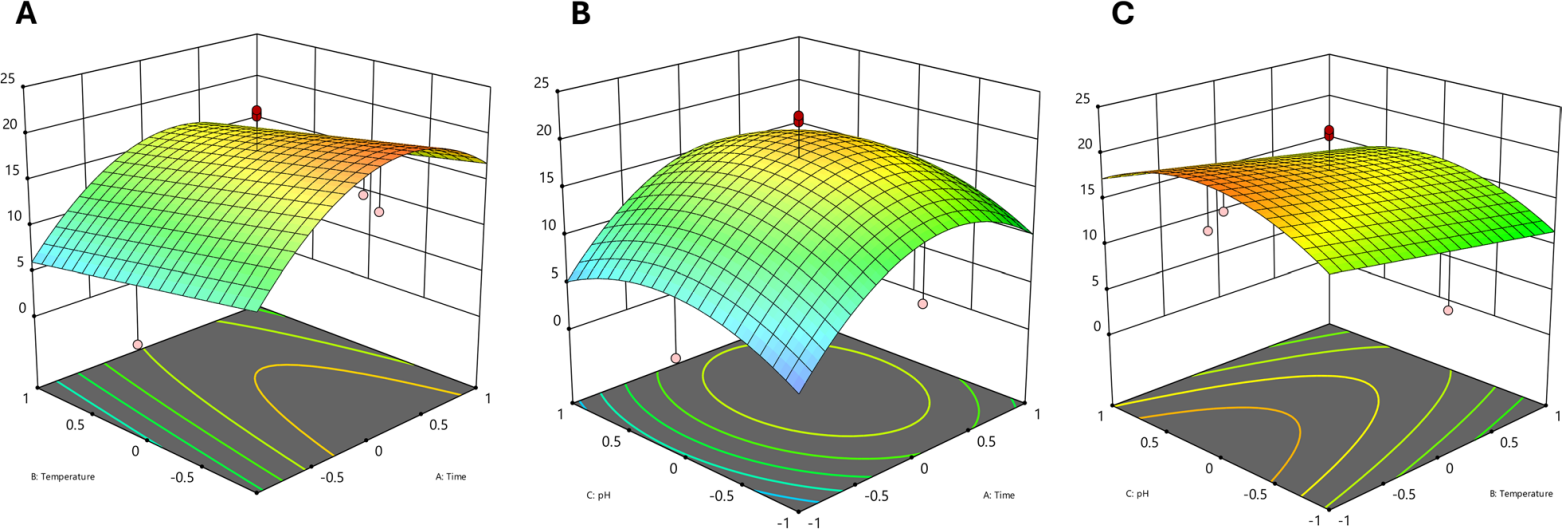
Plotting of response surfaces for the laccase production by *T. harzianum* AUMC14897: **A** changing the temperature and duration of fermentation; **B** changing the duration and pH; **C** changing the temperature and pH

Figure depicted a three-dimensional response surface graph for laccase production by *T. harzianum* AUMC14897, demonstrating the interactions of the three variables to identify the optimal amount of each variable for maximal response. Laccase synthesis was significantly impacted by the temperature and fermentation duration interaction, as demonstrated by Fig. 3A. Temperatures and fermentation times initially led to an increase in laccase production, which finally decreased with higher temperatures and longer fermentation times. This might be due to the nutrients in the medium are progressively depleted, affecting the fungi's physiology and resulting in the deactivation of the enzyme secretary mechanism. The selected fungal endophyte displayed significant laccase activity after just five days of cultivation, which is much shorter compared to the other study [52]**.**

As shown in Fig. 3B, laccase activity increased with increasing pH value and fermentation duration. Laccase activity decreased when the pH rose. Lower pH may alter fungal development, preventing laccase synthesis from being metabolized, which lowers laccase production [53]**.** These findings were comparable with prior studies on laccase synthesis by other fungus species, including *Fusarium solani* [53]**,** and *Ganodermasp. *[43]**. ‏**The findings showed that an optimum pH might increase laccase synthesis. Moreover, as in Fig. 3C, laccase synthesis rose when fermentation temperature increased at higher pH. In addition, Fig. 3C showed that laccase activity fell dramatically as temperature increased more. An increase in fermentation temperature caused sporulation, which in turn slowed the development of mycelia in fungus. As a result, it may induce a reduction in enzyme activity. Similar to our results, when fungi were grown at temperatures higher than 30°C, it was found that the activity of enzymes was reduced [9, 54]. ‏Finally, the ideal fermentation parameters for laccase production by *T. harzianum* AUMC14897 were determined as carbon source (sucrose 10g/L), nitrogen source (glycine 10g/L), fermentation duration of 5 days, temperature of 30 ^◦^C, and pH of 6. Notably, this is the first study for high laccase production from endophytic *T.harzianum* in contrast to minimal laccase production from soil *T.harzianum* [55]**.**

### Purification of *T. harzianum* laccase enzyme

**Fig. 4:**
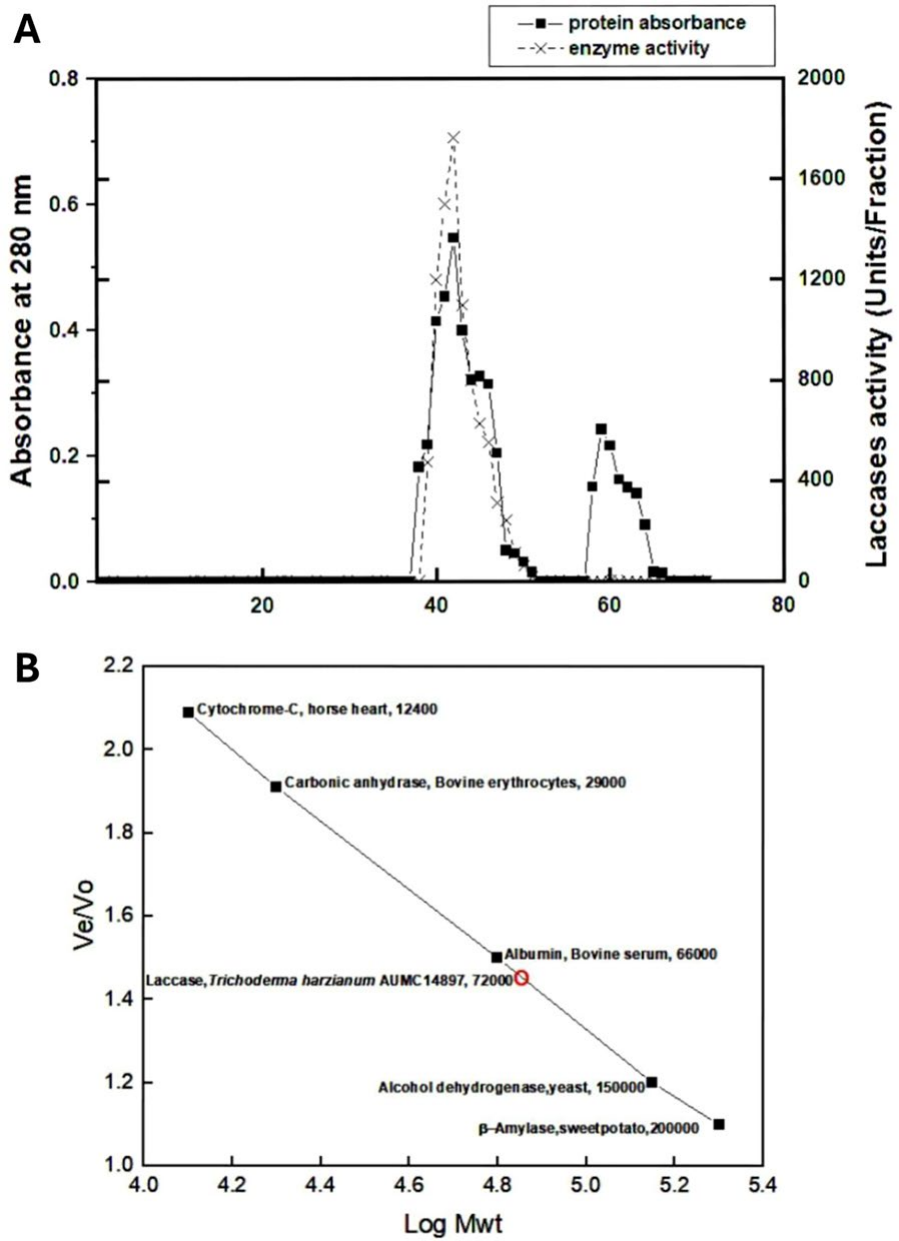
**A** *T. harzianum* laccase elution profile using Sephacryl 300 HR column. **B** Purified laccase's molecular weight from *T. harzianum* using Sephacryl 300HR (GFC) and standard protein markers which are cytochrome-C, horse heart (12.4 kDa), Carbonic anhydrase, Bovine erythrocyte (29 kDa), Albumin, Bovine serum (66 kDa), Alcohol dehydrogenase, yeast (150 kDa) and ß-Amylase, sweet potato (200 kDa)

Laccase produced by *T. harzianum* AUMC14897 in culture fluid under optimal growth conditions and medium composition underwent purification using multiple methods. The results of these purification procedures were detailed in Table 4. Following the use of 80% ammonium sulfate fractionation to precipitate the crude laccase enzyme, it was subjected to Sephacryl 300 HR gel filtration chromatography. Our findings revealed a significant 3.55-fold increase in specific activity of ammonium sulfate fractionation rising from 175.2 to 623.29 U/mg, with a recovery rate of 79.95%. Figure 4A illustrates the Sephacryl 300 HR gel filtration column, which displayed the highest specific activity at 2417.4 U/mg, indicating a 13.79-fold increase with a recovery rate of 66.44%. The eluted fractions from the Sephacryl column were collected and characterized based on the most effective purification fold. Previous studies have shown that laccase is a prevalent protein found in extracellular fluids. For instance, More et al. [56] reported the purification of extracellular laccase from *Pleurotus sp.* with activity of 2600 U/mg. Conversely, Mezaal et al. [57] found lower specific activity values compared to our results, with laccase from *Klebsiella pneumoniae K4* achieving a 10.8-fold increase to 7.52 U/mg protein with a 21.46% recovery rate. Additionally, laccase from *Lentinula edodes* SC-495 and *Panus tigrinus* 707 exhibited purification folds of 10.1-fold and 18.1-fold, with specific activities of 118.2 U/mg protein and 375.8 U/mg protein, and recovery rates of 13% and 16%, respectively [58]. Marino et al. [59] demonstrated that laccase from *Trametes versicolor* 11,269 had a specific activity of 397.7 U/mg protein with a recovery rate of 58.5% upon purification. Furthermore, enriched laccase from *Trametes orientalis*, a white rot fungus, was purified to a recovery of 47.33% and a specific activity of 20.667 U/mg [60].

**Table 4:** Purification table of laccase obtained from *T. harzianum*

Parameter	Steps				
	Total activity (U)	Total protein (mg)	Specific activity (U/mg)	Purification (fold)	Recovery (%)
Crude extract	11792	67.32	175.2	1	100
80% Ammonium sulphate fractionation	9428	15.126	623.29	3.55	79.95
Sephacryl 300 HR	7835	3.241	2417.4	13.79	66.44

By applying Sephacryl 300HR gel filtration chromatography with reference proteins, the molecular weight of the purified laccase from *T. harzianum* was determined to be 72 kDa (Fig. 4B). Hao et al. [61], Found that the GFC approach yielded a molecular weight of 66 kDa for the pure laccase obtained from the medicinal fungus *Agaricus blazei*, which was in close relation to our results. Additionally, according to [62], GFC was used to quantify the molecular weight of the pure laccase from the white rot fungus *Perenniporia tephropora*, which came out to be 63 kDa. Moreover, Al-soufi [63] discovered that using Superdex chromatography, the molecular weight of the laccase derived from *Fomitiporia mediterranea* was 60.8 kDa. Conversely, Park et al. [64] calculated the molecular weight of laccase from *Fomitella fraxinea* and *Basidiomycete* using Sephacryl S-200, which came out to be 47 kDa.

### Assessment of the purity and integrity of the purified laccase enzyme using SDS-PAGE and zymographic analysis

When proteins are exposed to SDS and a reducing substance that breaks disulfide bonds necessary for correct folding, the proteins unfold into linear chains that have a negative charge corresponding to the length of the polypeptide chain. Most fungal laccases are reported to have molecular weights between 60 and 90 kDa, of which 10 to 25% may be related to the degree of glycosylation [65]. Purified laccase from *T. harzianum* had a molecular weight of 72 kDa, according to Sephacryl 300HR (GFC) using standard proteins (Fig. 4B) and confirmed by SDS- PAGE electrophoresis.

**Fig. 5:**
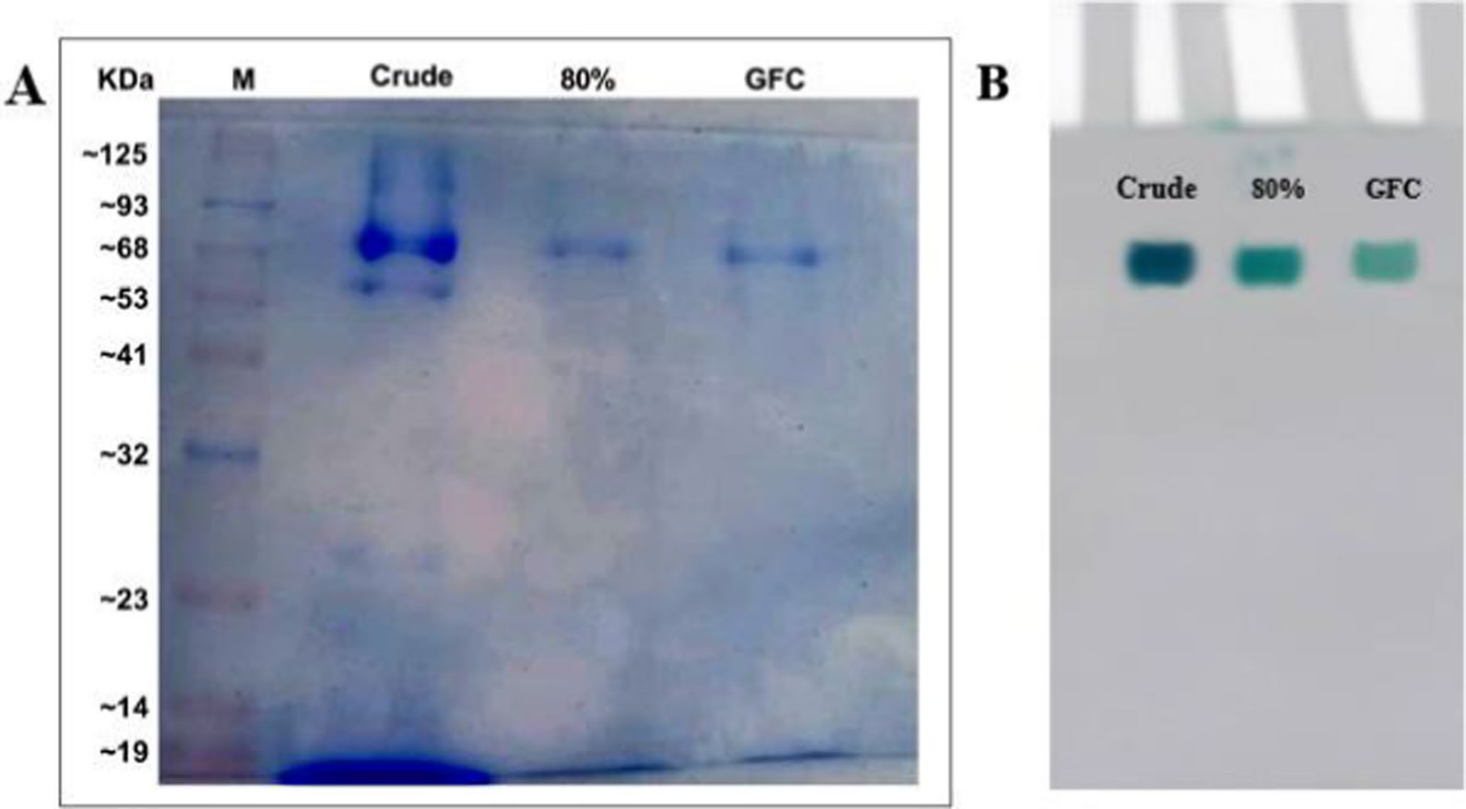
SDS-PAGE and Zymogram analysis of laccase purified from *T. harzianum*. **A** SDS-PAGE gel stained with Coomassie Brilliant Blue staining. **B** Native-PAGE gel stained with 1 mM ABTS. *(M: Protein Marker, 'Crude': The initial unpurified Lacasse, '80%': The fraction obtained after 80% ammonium sulfate precipitation and 'GFC': The final purified enzyme after gel filtration chromatography)

Figure 5A and Figure S5 Assessment the laccase enzyme purity and integrity through SDS-PAGE analysis. The well of gel crude lane shows the loaded enzyme crude extract, which is expected to contain various proteins, including laccase (72 kDa), and other proteins evidenced by multiple bands. The GFC Lane (gel filtration chromatography) exhibited a single band, signifying a high level of purity. Overall, the SDS-PAGE results confirm successful laccase purification steps through ammonium sulfate 80% fractionation and gel filtration chromatography, progressively reducing other proteins, for a highly purified isolated laccase enzyme and it was confirmed the molecular weight determined by Sephacryl 300 HR GFC method. The laccase's molecular weight in this investigation compared to that published by Edoamodu et al. [37], who concluded that laccase from *Enterobacter sp*. had a molecular weight equal to 75 kDa. Moreover**,** Chaoua et al. [66] estimated that molecular weight of laccase from *Trametes versicolor K1* was 68 kDa. Other research showed different molecular weight values as in purified laccase from *Arthrographis KSF2* showed a molecular weight of 55 kDa [67], and laccase from *T. atroviride* has molecular weight (57 kDa) [30].

Zymogram analysis is a powerful technique used to critical insights into enzymes molecular characteristics, optimal activity conditions, and potential applications. The diversity in molecular mass, isoforms, and biochemical properties among enzymes from different sources underscores their versatility and adaptability. In our study, the zymogram analysis visualized laccase with different band intensity correlating to the purifying levels after staining with 1 mM ABTS (Fig. 5B). Also, zymogram analysis elucidated that laccase isolated from endophytic *T. harzianum* has no isoforms as it appeared as a single smear band along the crude form, 80% ammonium sulphate fractionation and Sephacryl 300 HR column chromatography. Previous study confirmed our findings as showed by Aslam et al. [68] who proved that laccase from *Cladosporium cladosporioide* had single band in both SDS-PAGE and Native SDS.

### Characterization of the purified *T. harzianum* laccase

#### Effect of temperature and activation energy calculation (Ea)

**Fig. 6:**
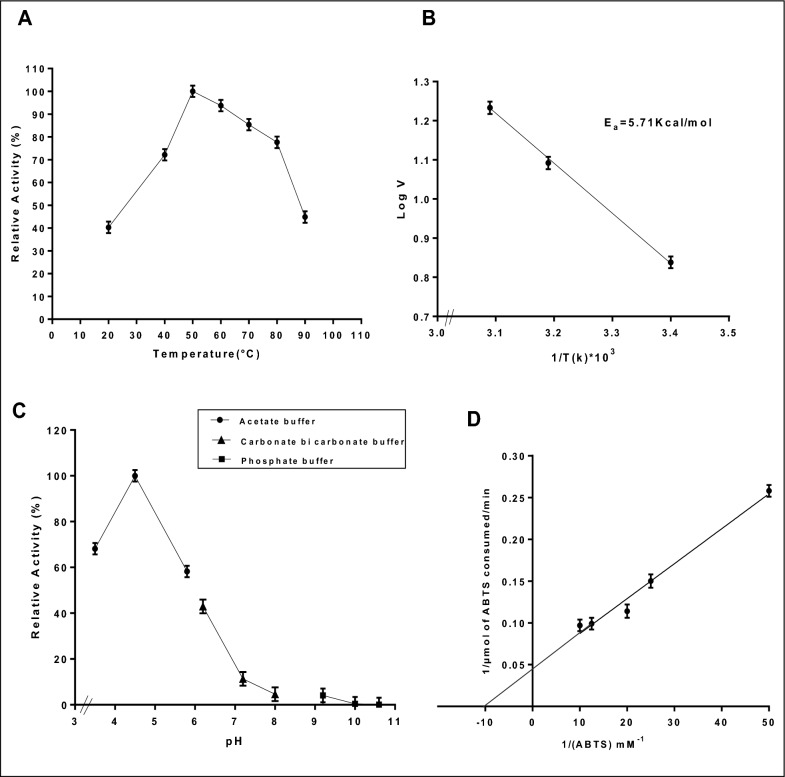
**A** Effect of different temperatures. **B** The activation energy (Ea) of purified laccase from *T. harzianum* using Arrhenius plot. **C** Effect of different pH values. **D** Line weaver-Burk plot for *T. harzianum* purified laccase under different ABTS concentrations

Temperature has an important environmental effect on conformation of enzymes as demonstrated in Fig. 6A, the purified *T. harzianum* laccase remained active over a temperature range between (20–90 °C). The laccase's optimum reaction temperature was noted at 50 °C, over this point, the activity gradually began rapidly to fall, reaching 44.8% at 90 °C. Other findings which were in line with our results observed that optimum temperature of purified laccase from *Ganoderma australe* was found to be 55 °C [69]. Also, Ezike et al. [27] observed that laccase from *Trametes polyzona* WRF03 had an optimum temperature at 55 °C. Moreover, Hao et al. [61], elucidated that the purified laccase from mushroom *Agaricus sinodeliciosus* had the highest activity at 50 °C.

The activation energy (E_a_) of the pure *T. harzianum* laccase was computed using the Arrhenius plot's slope, which demonstrated a linear change with temperature (Fig. 6B). The purified laccase's E_a_ for ABTS hydrolysis was 5.71 kcal/mol. This explained that *T. harzianum* laccase had high activity that sped up the reaction rate by reducing the activation energy required for it to initiate. Additional earlier research observed by Mateljak et al. [70], showed that purified laccase from *basidiomycete* had E_a_ equal to 3 kcal/mol. Further, Ezike et al. [27] stated that the activation energy of *Trametes Polyzona WRF03* laccase was 80.90 kJ/mol. Bakratsas et al. [71] stated that the activation energy of the pure laccase derives from *Pleurotus ostreatus* equal 20.0 kJ/mol.

### Effect of different values of pH and enzyme concentration on the activity of purified laccase

Many laccases derived from fungal sources exhibit activity in acidic or neutral pH ranges and become inactive in alkaline pH ranges. This explains how hydroxyl ions in an alkaline environment prevent electron transport between catalytic sites and block the T2 and T3 copper domains [72]. The pure laccase activity from *T.harzianum* had 100% relative activity at pH 4.5 as shown in Fig. 6C, on the contrary the activity of purified *T.harzianum* laccase declined at alkaline conditions with relative activity 0.109% at pH 10.6. Hence, it was revealed that the laccase from *T. harzianum* was acidophilic. Previous studies as Kumar et al. [73], elucidated that purified laccase isolated from *Aspergillus flavus* showed high activity at pH 4.5. Moreover, laccase isolated from *Pleurotus sajor-caju* MTCC 141 had high activity at pH 4.5 [74]**.** Furthermore, at pH 4 and 5, *the Alcaligenes faecalis* enzyme's NYSO laccase remained extremely stable, keeping over 92.4% and 100.3% of its initial activity, respectively [27].

Under standard conditions, the impact of varying enzyme concentrations on enzyme activity was investigated. As shown in the supplementary file (Fig. S3), the result demonstrated that a successive increase in laccase activity as the enzyme concentration increased per reaction mixture, from (0.045–61 U/mg), where the maximum enzyme activity was achieved. Previous findings were in line with our results as *Trematosphaeria mangrovei* laccase [31] and laccase from *Kluyveromyces* sp. Dw1 [75].

### Determination the kinetic parameters (K_m_ and V_max_ values)

Laccase's kinetic parameters from *T.harzianum* AUMC14897 exhibited high affinity (K_m_) for its substrate (ABTS) which equal to 0.1 mM and V_max_ 22.22 µmol/min as mentioned in Fig. 6D**.** Previous findings stated that laccase from *Marasmius sp* exhibits V_max_ of 1.45 mM/min and K_m_ constant value of 4.11 mM. [76]. Moreover, laccase isolated from a new strain of *Fomes fomentarius* exhibited high V_max_ values (60 mM/min) and low K_m_ (1 mM). Riffat et al. [77]**,** Ezike et al. [27], mentioned that by using ABTS as the substrate, the laccase from *Trametes polyzona* WRF03 has (K_m_ and V_max_) equal to 8.66 μM and 14.29 μmol/min, respectively. The affinity of *Sphingobacterium ksn-11* purified laccase for ABTS was significantly higher (K_m_ 2 mM &V_max_ 33 U.mg^−1^) as mentioned in Neelkant et al. [78].

### Half life span of purified *T. harzianum* laccase

**Fig. 7:**
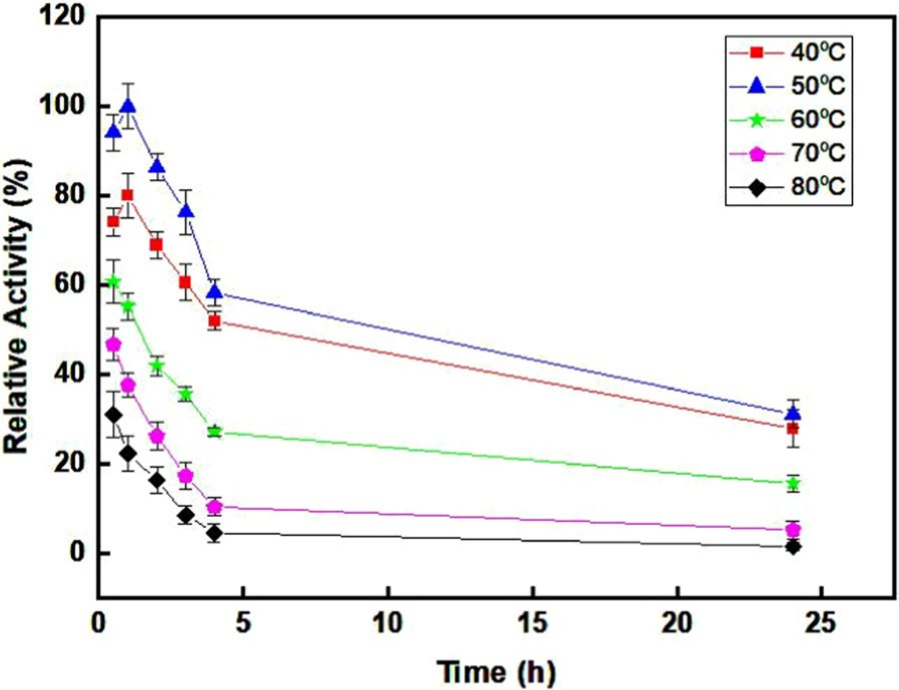
Half life span of purified laccase from *T.harzianum*

The purified laccase from *T. harzianium* exhibited a half-life of 4 h at 50 °C, measured concerning its enzyme activity at the optimal temperature. Remarkably, even after 24 h at 50 °C, the enzyme retained over 30% of its activity (Fig. 7; Fig.S6). This half-life range aligns with previous studies; for instance, the laccase enzyme from *T. hirsuta* demonstrated a half-life of 5 h at 65 °C [79]. Similarly, Navada et al. [28] reported that the purified laccase from *T. hirsuta* remained stable for 6 h at 50 °C. In contrast, a study on recombinant laccase from *Trametes trogii* BAFC 463, revealed a significantly shorter half-life of 45 min at 70 °C [42].

### Effect of different metal ions and EDTA chelating agent

The presence of heavy metals in wastewater causes a complex challenge that extends beyond direct environmental and health impacts and could significantly interfering with enzymatic processes crucial for dye removal and wastewater treatment. Thus, it is imperative to develop a remediation technique that is economical, simple, effective, and eco-friendly, while also considering the intricate interactions between heavy metals and enzymes [80]. In Table 5, Mg^2+^ at 1 mM concentration activated purified laccase (relative activity: 120%), but at 2 mM, it displayed significant inhibition (relative activity 43%). Mono, di, and trivalent cations K⁺, Cu^2^⁺and Al^3^⁺exhibited high laccase activity at 2 mM concentration, with relative activity of 74.9%, 83.8%, and 73.9%, respectively. However, divalent cations such as Ca^2^⁺and Hg^2^⁺ inhibited laccase activity at 2 mM concentrations, with relative activity of 37.17% and 41.6%, respectively. Additionally, the presence of EDTA (1 mM, 2 mM) completely inhibited laccase activity, yielding a relative activity 3.33% at 2 mM concentration.

**Table 5:** Effect of different metal ions and EDTA inhibitor on activity of purified laccase from *T.harzianum*

Metal ions/inhibitors	Relative activity (%)	
	1 mM	2 mM
Control (without metals inhibitors)	100	
Ca^2^⁺ (CaCl_2_)	47.7	37.17
Mg^2⁺^(MgCl_2_)	120	43
Hg^2⁺^(HgCl_2_)	54.09	41.6
Cu^2^⁺ (CuSO_4_)	67.8	74.9
Al^3^⁺ (AlCl₃)	79.5	83.8
K⁺ (KCl)	63.2	73.9
EDTA	5.39	3.33

Metals as Mg^2+^, K⁺, Cu^2^⁺and Al^3^⁺ increase the activity of pure *T. harzianum* laccase due to their involvement in the catalytic process of the enzyme reaction and play an important co-factor for it. Conversely, some metals such as Ca^2+^ and Hg^2+^ tightly bind to the laccase T1 site and operate as competitive inhibitors for e-donors, blocking substrate access to the T1 site and preventing e-movement to the T1 active site. Laccase activity is inhibited as a result of this action. Additionally, as demonstrated by the noncompetitive inhibition model, they can cause the enzyme to undergo conformational alteration and promote the breakdown of the trimer complex, which consists of the substrate, enzyme, and metal ion. [81]. Our findings are consistent with previous research, particularly the study by Si et al. [69], which examined laccase isolated from the white rot fungus *Trametes hirsuta*. Their research showed that, Cu^2+^, Mg^2+^, and K^+^ ions enhanced laccase activity while Ca^2+^ and Hg^2+^ ions inhibited laccase activity. However, our results differ from Si et al. in one key aspect as their study found that Al^3+^ inhibited laccase activity, our research showed that Al^3+^ actually enhanced laccase activity. This comparison highlights both the similarities in laccase behavior across different fungal species and the potential for species-specific variations in enzyme response to metal ions. Also, our findings align with earlier study on laccase from *Arthrographis ksf* that showing inhibition by Ca^2+^and Hg^2+^ while being activated by Cu^2+^ [67]. In contrast, some reports, like that of laccase from *P. ostreatus* strain 10969, indicate inhibition by copper ions [78]. Atalla et al. [31], noted that *Trematosphaeria mangrovei* derived laccase enzyme retained about 64.38% and 57.58% of its initial activity in the presence of Mg^2+^ and Hg^2+^, respectively. While the other metal ion, Ca^2+^, had minimal impact on the activity of enzyme, the laccase enzyme maintained roughly 71% of its initial activity when K^+^ was present.

Our study also examined the effect of EDTA on purified laccase activity with concentration (1, 2 mM), which revealed the totally inhibition effect on purified laccase activity. Our findings agreed with those of Liu et al. [82], who documented that EDTA had an inhibitory effect on the activity of pure laccase. It might be the result of type I copper ions chelating, which would suggest that these ions are involved in the catalytic reaction of laccase. Furthermore, type II copper ions were probably made easier to collect by EDTA, which further reduced laccase activity [83]. In contrast to our study Castano et al. [84], mentioned that EDTA did not show a significant reduction in activity of purified laccase from the native fungus *Xylaria sp*.

### Wastewater treatment

#### Synthetic dye decolorization

The chemical structure of dyes plays a major role in their decolorization efficiency. Anthraquinone, thiazine, triphenylmethane, and azo are examples of synthetic dyes that are linked to different industries and are among the most used types of dyes. These synthetic dyes are often resistant to conventional wastewater treatment methods and can persist in the environment and cause serious health problems in both humans and animals. However, certain organisms, particularly white-rot fungi, have demonstrated the ability to degrade these complex aromatic structures. Decolorization of dyes with enzymes was a very efficient, environmentally sufficient, and economically competitive method of decomposing these toxic dyes [85].

**Fig. 8:**
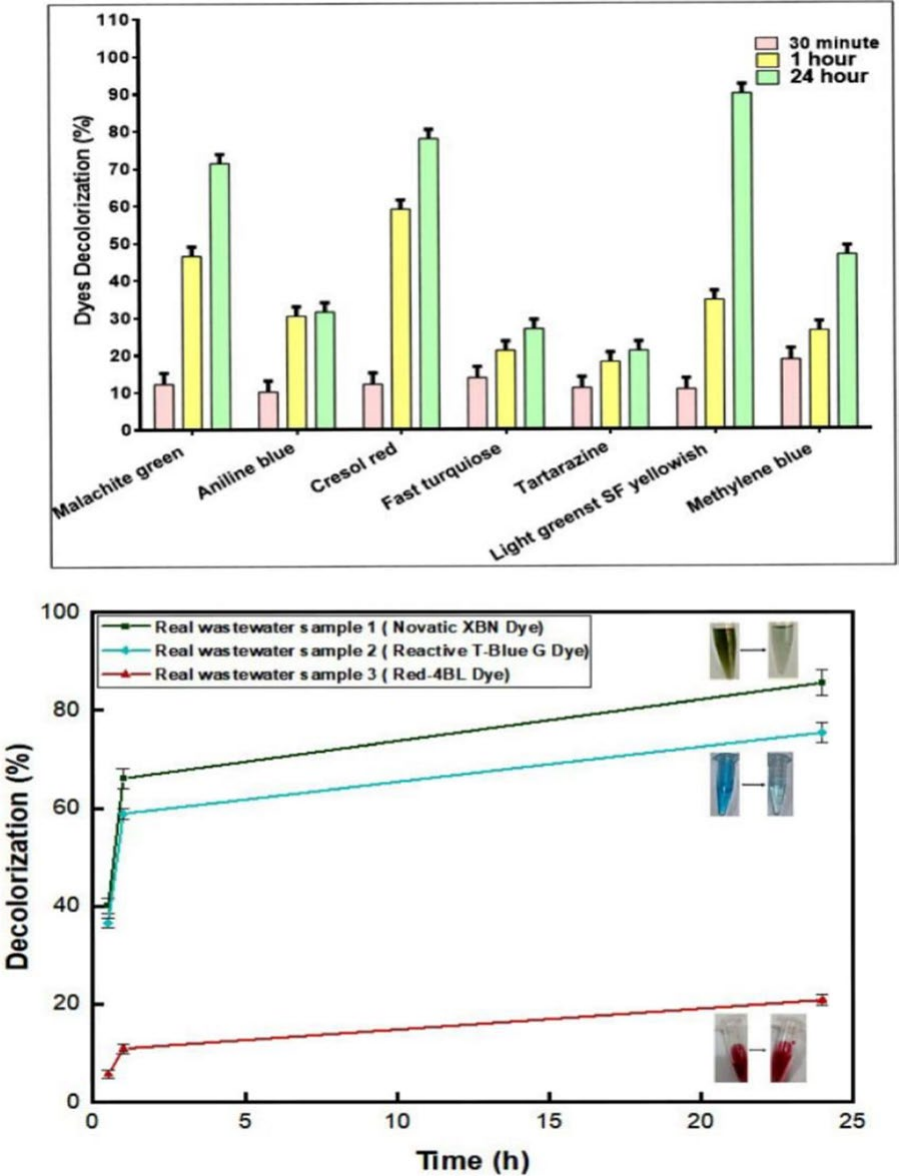
**A** Synthetic dye decolorization effect via pure laccase from *T.harzianum* AUMC14897. **B** Real dye decolorization impact by using purified laccase from *T.harzianum* AUMC14897

Under optimum conditions of pH 4.5 and 50 °C, purified laccase obtained from *T. harzianum* AUMC14897 (20 U/mL) was incubated with several types of dyes as demonstrated in Fig. 8A. Our results revealed that tri aryl methane, light green SF yellowish was decolorized quickly, reaching the highest extent of 89% following a 24-h incubation period. Additionally, it was shown that the laccase had a potent capacity to decolorize malachite green and cresol red, with 71.4, 77.89% respectively, and moderate decolorization capacity towards thiazine dye as methylene blue (50 mg/L) and triphenylmethane dye as aniline blue, with decolorization percentage of 46.75 and 31.57%, respectively, after incubation for 24 h. Meanwhile, azo dye, tartarazine, and phthalocynaine dye as fast turquoise blue, the laccase showed quite minimal effect with decolorization capacity 21.1 and 26.93% respectively.

Various dyes did not undergo the same degree of decolorization, which could be attributed to variations in their redox potentials and the compatibility of their steric structures with the enzyme's active site [86]. Triarylmethane as light green SF yellowish is a class of dyes that are mostly employed in the dying and textile industries. This dye is extremely poisonous and have a major environmental concern in recent years. In addition, it has the capacity to accumulate and penetrate through skin contact, and if swallowed or breathed, it can irritate. In previous research was in line with our results point of view was conducted by Schiller et al. [87] who observed that laccase was responsible for the N-demethylation that decolorized triphenylmethane and triarylmethane dyes.

Tartrazine is one of the azo dyes that require a longer time to decolorize because they contain one or more azo groups (-N = N-) in their chemical structures. These dyes are known to be resistant to enzymatic decolorization, have good photolytic stability, and are resistant to major oxidizing agents. Thus, laccase has minimal effect on the breakdown of azo-dye structures but with the help of peroxidase in the presence of H_2_O_2_ can oxidize their chromophore assemblies with subsequent generation of free radicals [88].

Phthalocyanine dye is an aromatic, macrocyclic, organic compound with the formula (C_8_H_4_N_2_)_4_H_2_. It comprises four iso-indole units linked by a ring of nitrogen atoms (fast turquoise blue). Since fast turquoise blue is a copper-containing dye, its poor decolorization effectiveness can be explained by the fact that the transformation cannot dissolve the metal-ion coordination bond.

Furthermore, thiazine dye comprises three organic compounds of the heterocyclic series. One of the most common thiazine dyes is methylene blue. Due to its high water solubility, it can be dissolved in water to form a stable solution at room temperature. Methylene blue's chromophore group is the N–S conjugated system on the aromatic heterocycle in the middle. The core aromatic ring of methylene blue is broken first, followed by the side aromatic rings. The fragments created by these two stages are subsequently converted into intermediate species, such as R-NH^3+^, phenol, and aniline, during the enzymatic degradation by laccase. Our results were aligning with the previous studies of Ghobadi Nejad et al. [89]**,** who showed that *Phanerochaete chrysosporium* laccase own efficient decolorizing activity (100%) toward malachite green and moderate decolorization was also observed for methylene blue. Further, *T. harzianum* laccase demonstrated decolorization up to 49.87% and 46.15%, respectively, against methylene blue and malachite green [90]. Additionally, dye biodegradation by laccase from *Trichoderma harzianum* M06 are presented maximum removal of cresol red reached to (88%) [91]. Also, *Trametes polyzona* WRF03's purified laccase decolorized malachite green with a high efficiency of 78.4%, while it decolorized methylene blue with a low efficiency of 0.38%. [27]. Further previous findings mentioned that decolorization efficiency of *ZrF (Zirconia-fucoidan)* laccase reached to 60% of tartrazine dye [92].**‏** The purification procedures used, the enzyme's unique catalytic efficiency, the dyes' structural characteristics, and the concentration utilized could all be contributing factors to the variations in decolorization rates observed in the numerous studies. All laccase dyes decolorization mechanisms were performed in the supplementary file (Fig. S7 to Fig S15).

### Environmental real wastewater samples dye decolorization

Laccase derived from *T. harzianum* AUMC14897 (20 U/mL) was able to decolorize three real wastewater samples containing Novatic green XBN (56.83 mg/L), Red 4BL (57.80 mg/L) and Reactive T. Blue G (49.79 mg/L) dyes (Fig. 8B, FigS4). Vat dyes as Novatic XBN are water-insoluble, however they have two or more keto groups (> C = O) divided by a conjugated double bond system, which is used in the vatting process to transform them into alkali-soluble enolate leuco compounds (> C—O −) [93]**.** Our results elucidated that the purified *T.harzianum* laccase showed high decolorization percentage towards Novatic green XBN reached to 85.60% at 50 °C after 24 h. Other previous findings mentioned by Mohanty and Kumar [94] observed that Vat Green XBN dye decolorization efficacy by laccase enzyme could reached 94.96% within 20 h.

Moreover, our findings elucidated that the purified *T.harzianum* laccase showed good ability towards decolorization of Reactive T. Blue G reached 75.40% at 50 °C after 24 h. Comparable previous results mentioned that *Cyathus bulleri* laccase was engineered to degrade effectively reactive T. blue with 78 to 95% decolorization percentage in 30 min [95].

Azo dyes as Red-4BL are recognized to be resistant to enzymatic decolorization and hence require more time for dye decolorization [96]. The results of pure *T.harzianum* laccase investigated low decolorization percentage towards Red 4BL with decolorization percentage 20.80% at 50 °C after 24 h. our study was aliens with the previous laccase produced by *P. ostreatus* IBL-02 which observed 100% decolorization to Drimarine blue textile dye effluent after 5 h and low dye decolorization was observed towards Red 4BL dye under optimum conditions [97].

Hence, our findings confirmed that the catalytic dye decolorization efficacy of the investigated laccase from *T.harzianum* AUMC14897 permitted its wide applicable usage in wastewater treatment.

## Conclusion

The use of bioremediation techniques in the degradation of industrial pollutant dyes is a hotly pursued research topic. Therefore, this study sheds light on the promising application of Laccase from a Novel Endophytic *T. harzianum* AUMC14897 in wastewater treatment, by emphasizing its potential for efficient dye decolorization. The Endophytic *T. harzianum* AUMC14897 was optimized to obtain ideal fermentation parameters for laccase production as carbon source (sucrose 10 g/L), nitrogen source (glycine 10 g/L), fermentation duration of 5 days, temperature of 30 °C and pH 6. Moreover, The purified laccase appeared as a single protein band with a molecular weight of 72 KDa in SDS PAGE. Also, Laccase from Endophytic *T. harzianum* AUMC14897 didn’t observed any isozymes in zymogram analysis*.* Furthermore, Endophytic *T. harzianum* AUMC14897 pure laccase was thermostable at 50 °C at a wide time interval, and it was acid tolerant at pH 4.5. Besides, it is worth mentioning that the pure Endophytic *T. harzianum* AUMC14897 laccase is highly effective in decolorizing several hazard dyes effluent from industrial factories. The highest laccase decolorization efficiency after 24 h was for malachite green, cresol red and light greenst SF yellowish dyes. According to our understanding, this is the first report of a thermostable acidophilic laccase produced novel fungal endophyte *T. harzianum* AUMC14897 that was initially isolated from cladodes of *Opuntia ficus-indica* and we recommended using it in many practical environmental remediations.

## Further directions

Based on our results, further research could focus on immobilization of Laccase from Endophytic *T. harzianum* AUMC14897, identification of intermediate degradable products of dyes to ensure its complete mineralization. Also, investigate the enzyme reusability under various environmental conditions.

## Supplementary Information

Supplementary material 1.

## Data Availability

The data was available with the corresponding author upon request.

## Abbreviations

Mg: Milli gram
U: Unit
ABTS: 2,2'-Azino-bis(3-ethylbenzothiazoline-6-sulfonic acid)
PDA: Potato dextrose agar media
EDTA: Ethylene di amine tetra acetic acid
BOD: Biochemical oxygen demand
COD: Chemical oxygen demand
BLAST: Basic local alignment search tool
GFC: Gel filtration chromatography
SDS-PAGE: Sodium dodecyl sulfate–polyacrylamide gel electrophoresis
K_m_: Affinity constant
V_max_: Maximum enzyme velocity
E_a_: Activation energy
