# Supplementary material for: Optimization, purification and characterization of laccase from a new endophytic Trichoderma harzianum AUMC14897 isolated from Opuntia ficus-indica and its applications in dye decolorization and wastewater treatment
Source: Microb Cell Fact. 2024 Oct 5;23:266. doi: 10.1186/s12934-024-02530-x (PMC11453076; doi:10.1186/s12934-024-02530-x)
Supplement: Supplementary file 1 — Supplementary material 1. [file 12934_2024_2530_MOESM1_ESM.pdf]

<u>Supplementary file</u>

Optimization, Purification and Characterization of Laccase from a new Endophytic *Trichoderma harzianum* AUMC14897 isolated from *Opuntia ficus-indica* and its applications in Dye Decolorization and Wastewater Treatment

Maha M. Salem[1]*, Tarek M. Mohamed[1], Aya M. Shaban [1], Yehia A.-G. Mahmoud[2], Mohammad A. Eid[2], Nessma A. El-Zawawy[2]

[1]Biochemistry Division, Chemistry Department, Faculty of Science, Tanta University, Tanta, Egypt.

[2]Botany and Microbiology Department, Faculty of Science, Tanta University, Tanta, Egypt.

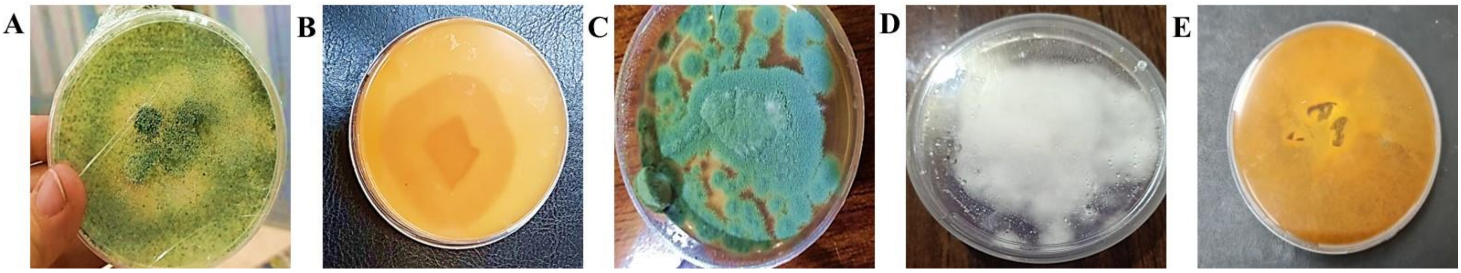

Figure (S1). Fungal endophytes isolated from *O. ficus-indica* and screening of Lacasse activity. A. F-1 strain, B. Positive Lacasse activity of F-1 strain, C. F-2 strain, D. F-3 strain, E. Negative Lacasse activity of F-2 and F-3 strains.

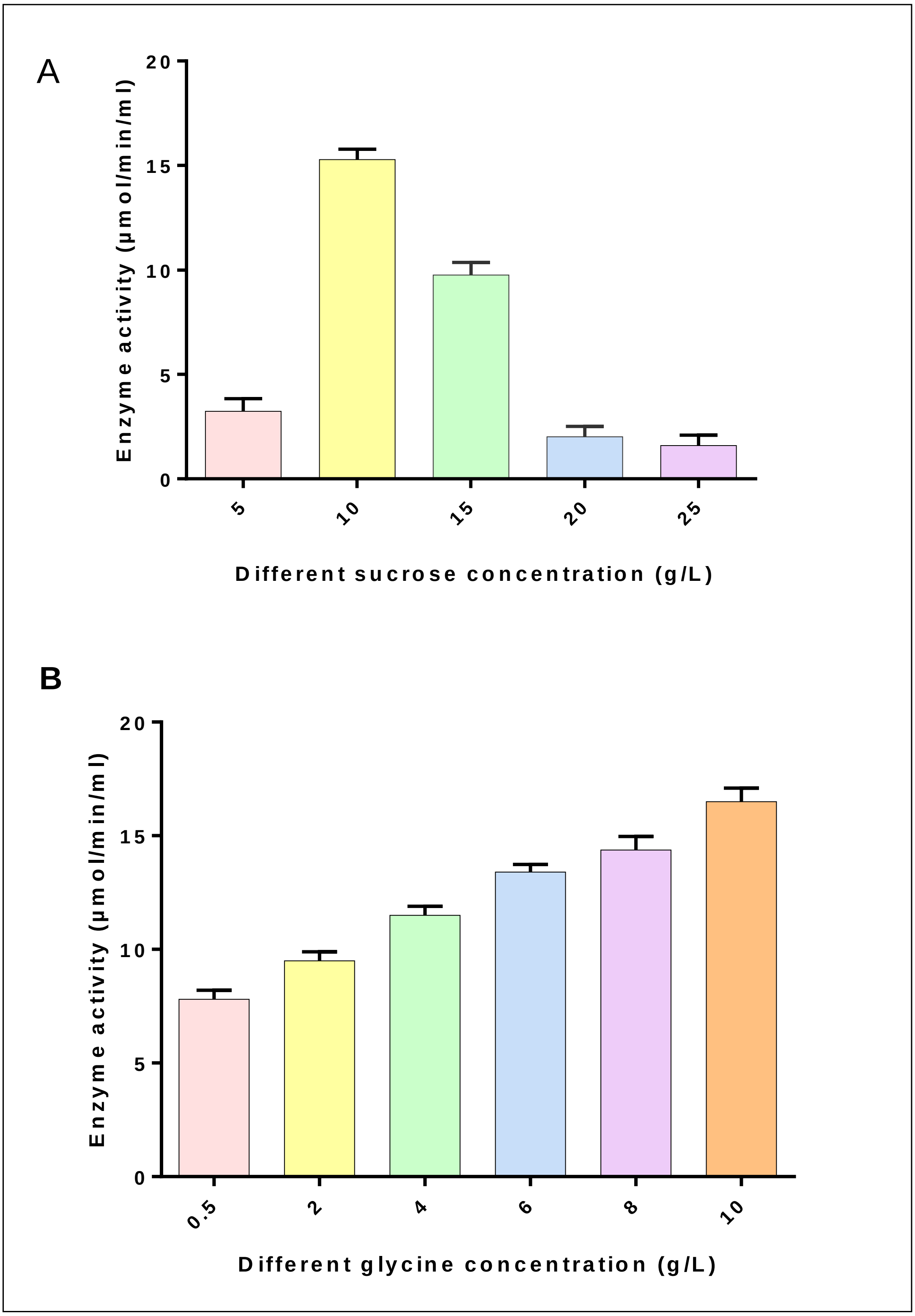

Figure (S2). Effect of nutrients on laccase production by *T. harzianium* AUMC14897 **(A)**. Different sucrose concentration. **(B).** Different glycine concentration for laccase production from *T.harzianum*

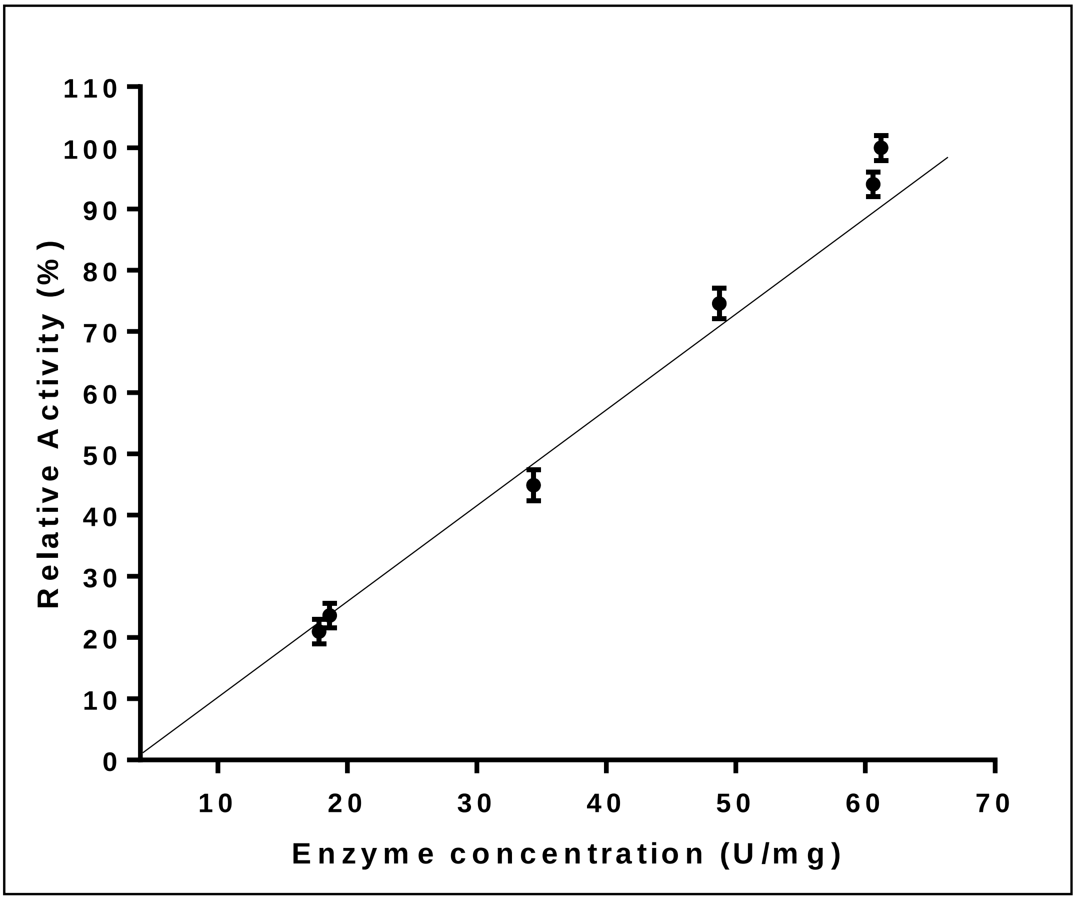

Figure (S3). Effect of different enzyme concentration on purified laccase from *T.harzianum*.

Table S1. Synthetic and environmental real wastewater dyes used in this study

Dyes	Molecular structure	Molecular Weight (g/mol)
**Light green SF yellowish (εmax= >600 at 631-634 nm in water)**	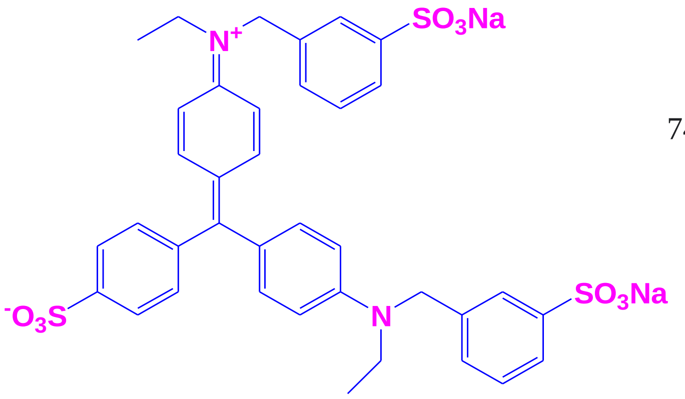	749.893
**Cresol red (εmax= 442.01 - 590.16 nm)**	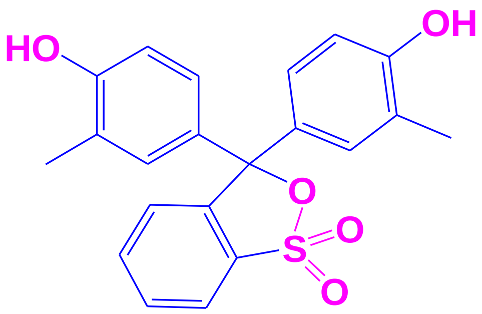	404.41
**Malachite green (εmax= 617 nm)**	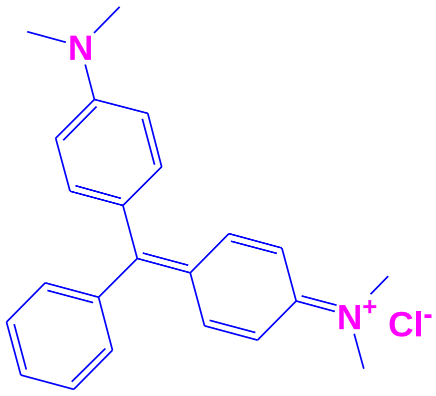	364.91
**Aniline blue (εmax= 594 – 610 nm)**	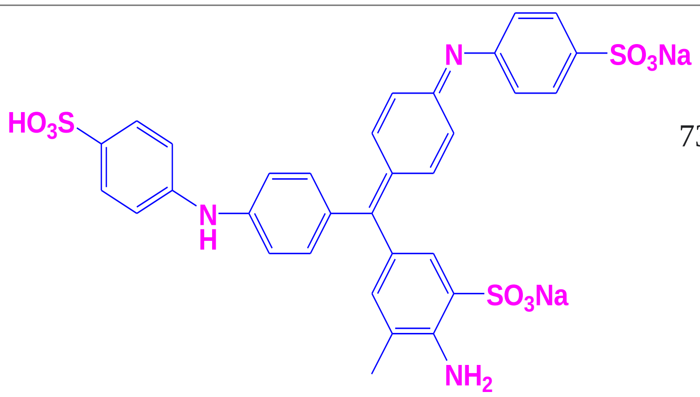	737.72

**Tartarazine (εmax= 435-500 at 425-429 nm in water)**	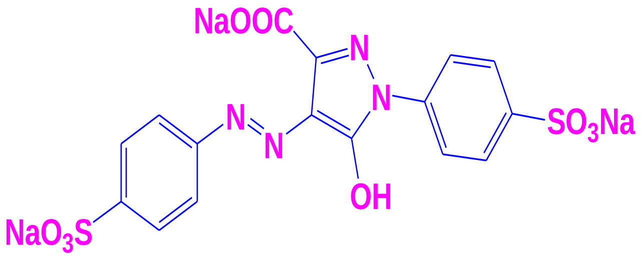	534.3
**Fast turquoise blue (εmax= 550 nm)**	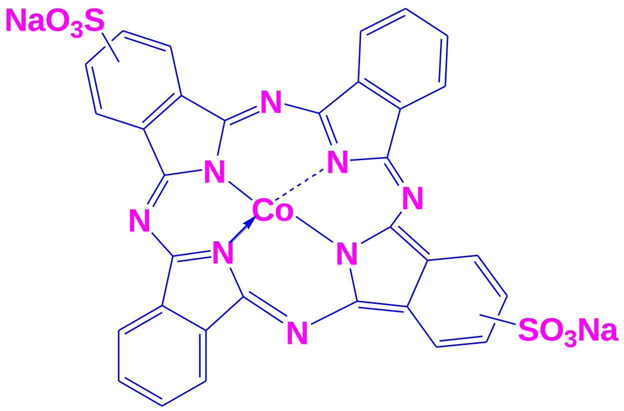	780.17
**Methylene blue (εmax= 665 nm)**	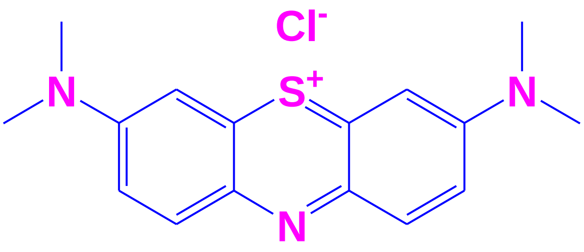	319.85
**Novatic green XBN (εmax= 527 nm)**	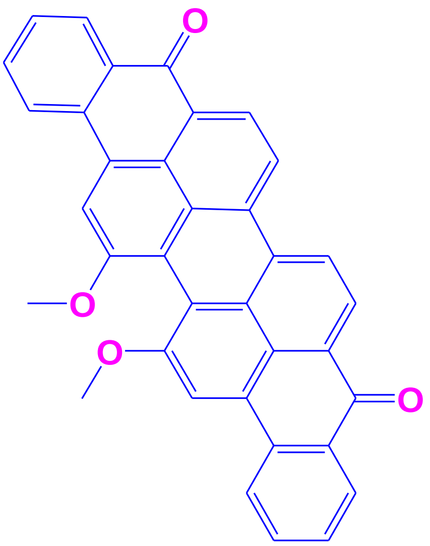	516.64

**Red 4BL** (**εmax= 655 nm**)	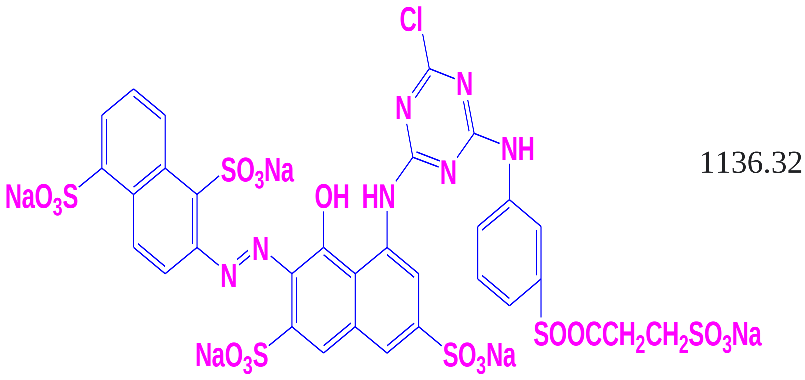	1136.32
**Reactive T. Blue G** (**εmax= 555 nm**)	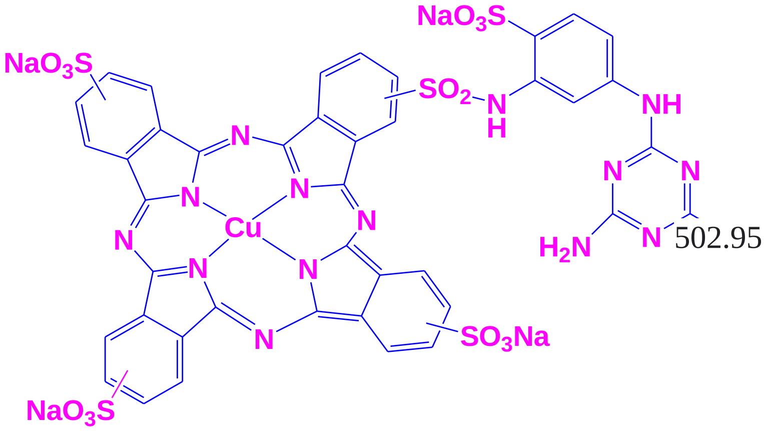	502.95

Table S1 Continued

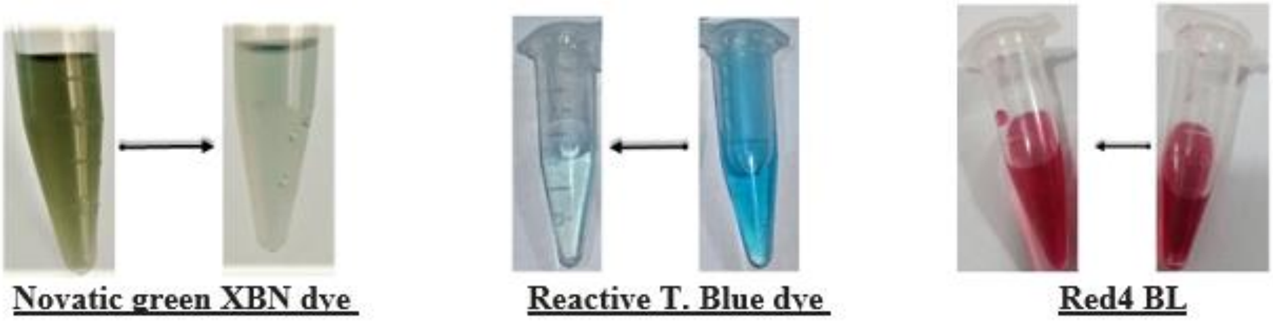

Figure S4: The images for real wastewater dye samples before and after 24h treatment with laccase

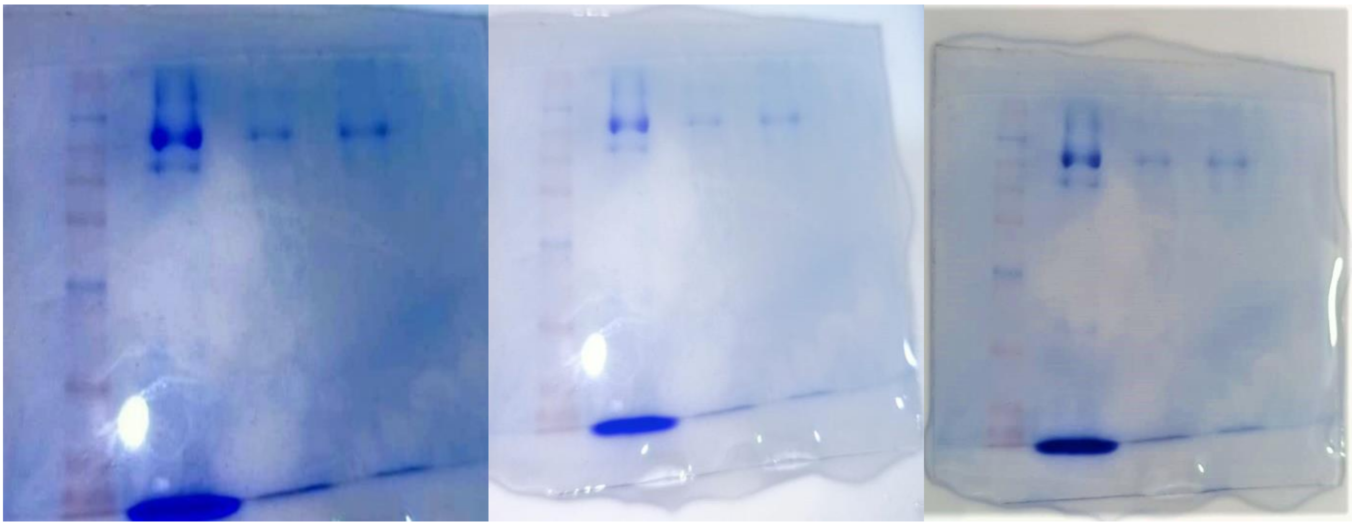

Figure S5: The raw images SDSPAGE for laccase different purification steps

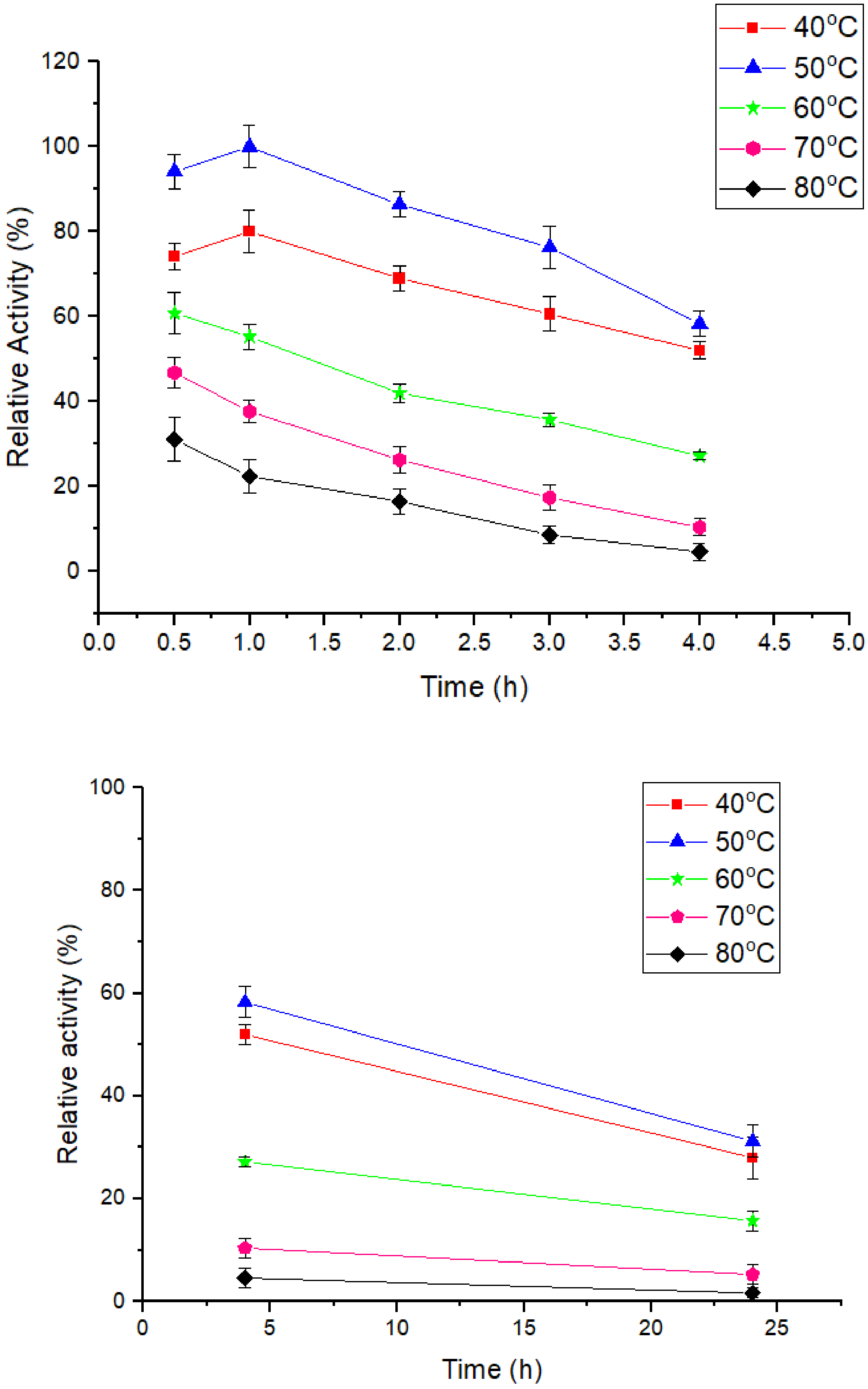

Figure S6: The analysis emphasizes the initial rapid changes in relative enzyme activity during the first 5 hours, followed by a plateau over the next 20 hours.

Mechanisms of laccase for dye decolorization used in our study

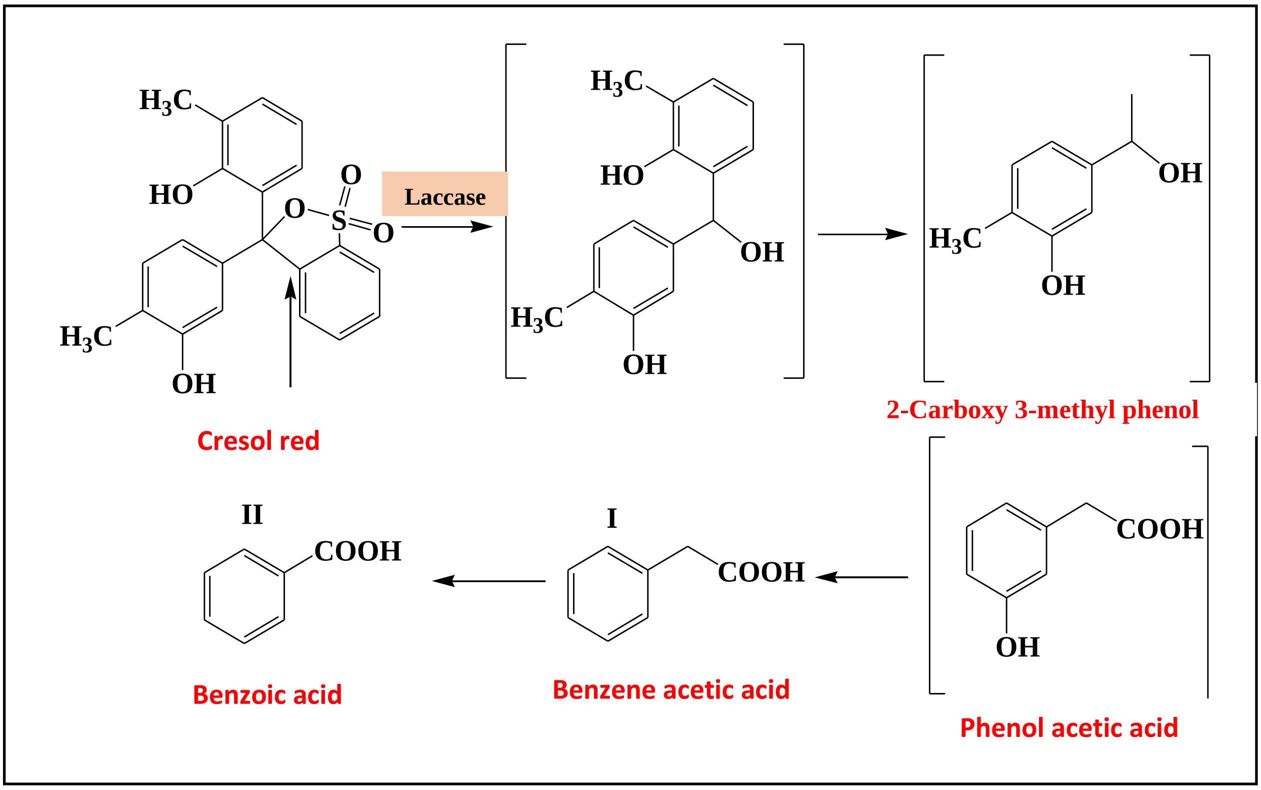

Figure S7. Degradation of cresol red by laccase

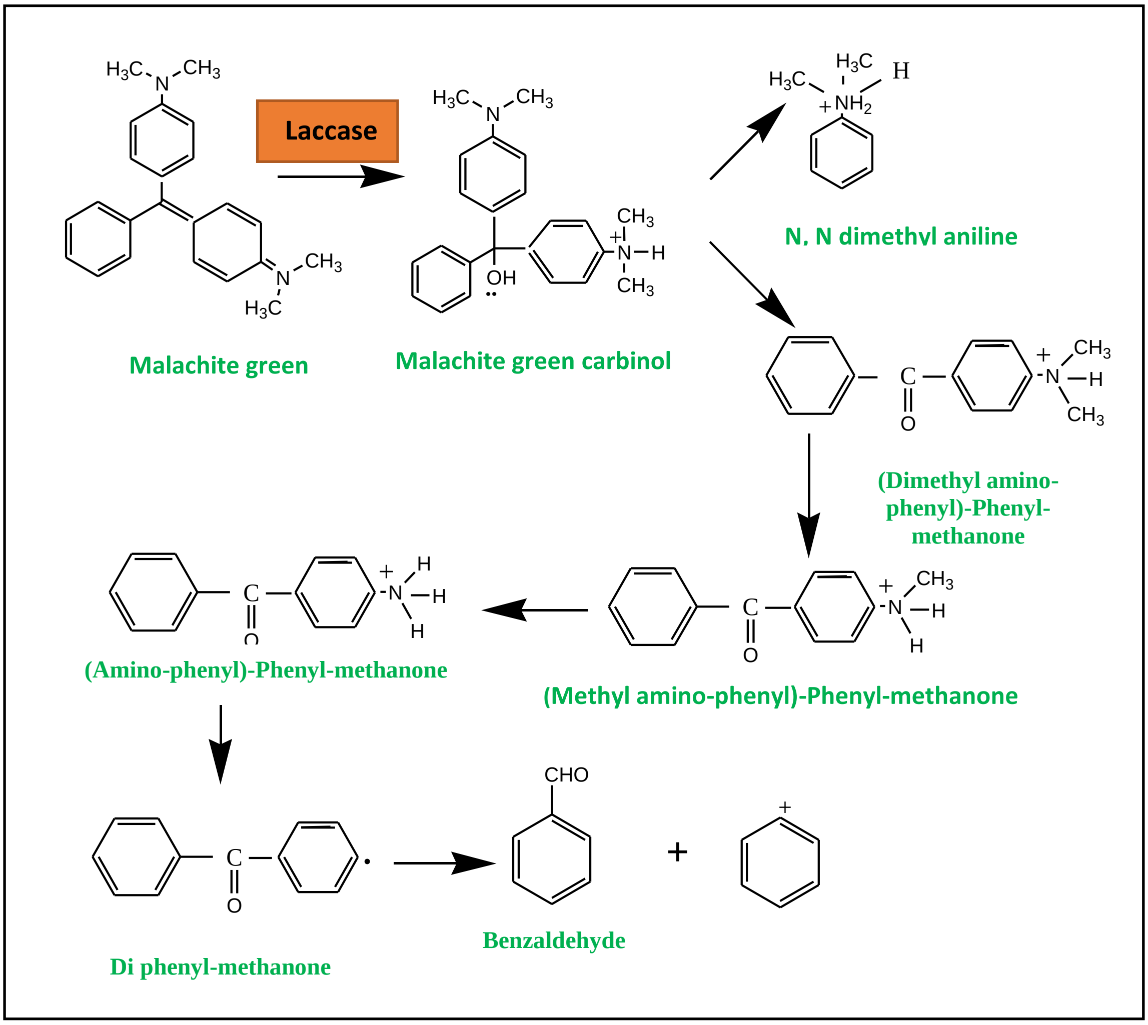

Figure S8. Degradation of malachite green dye by laccase

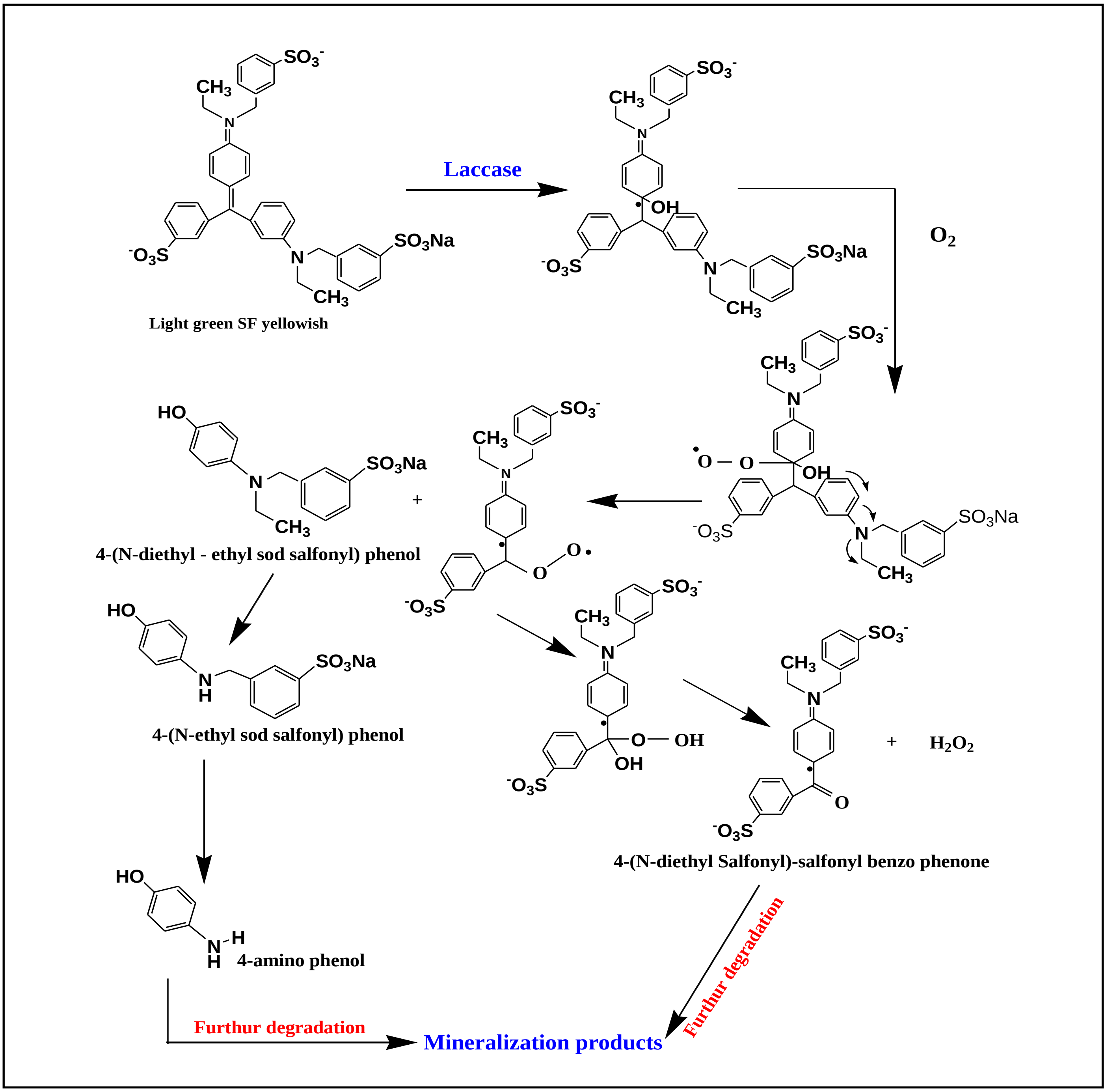

Figure S9. Degradation of light green SF yellowish dye by laccase

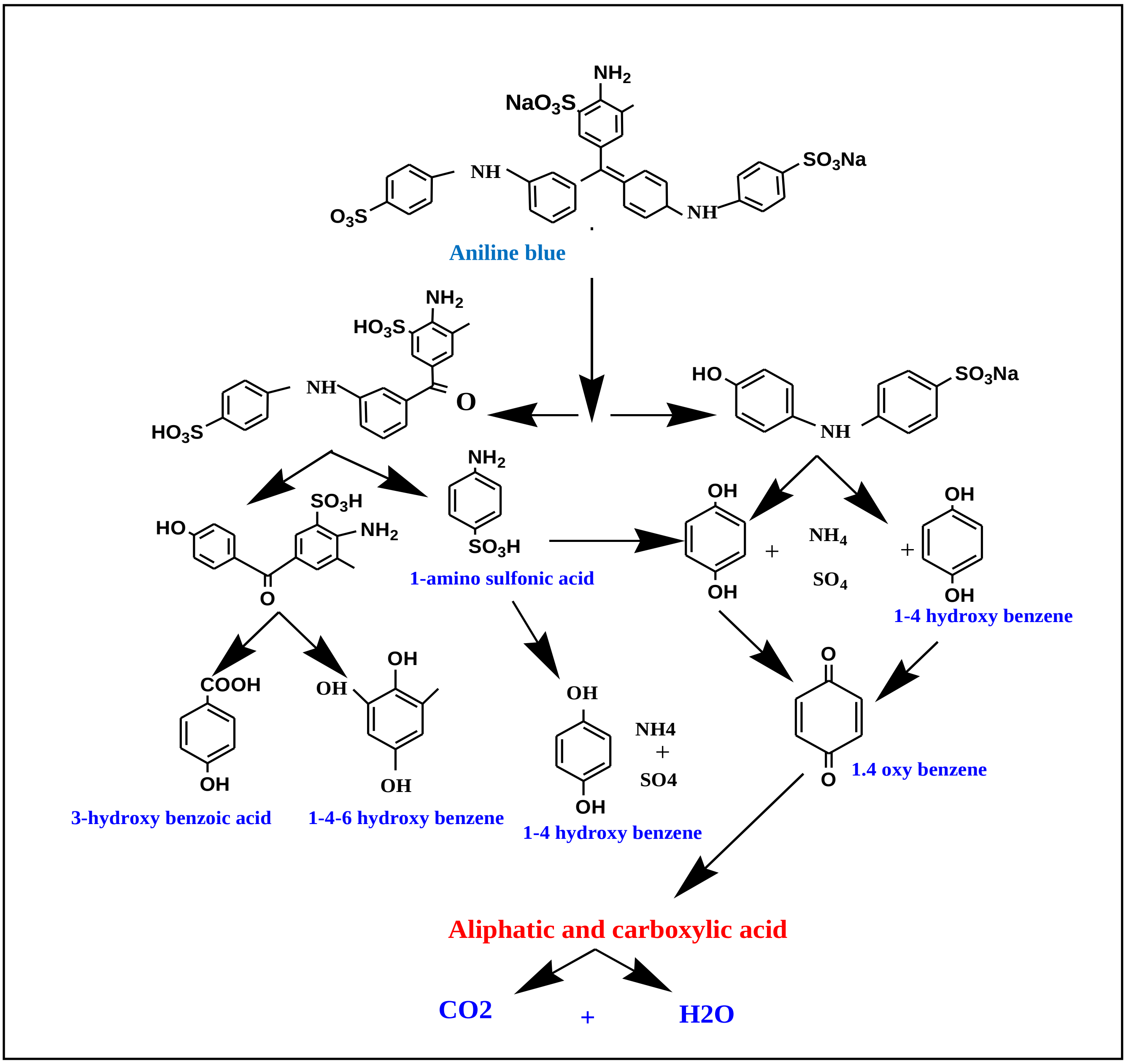

Figure S10. Degradation of aniline blue dye by laccase

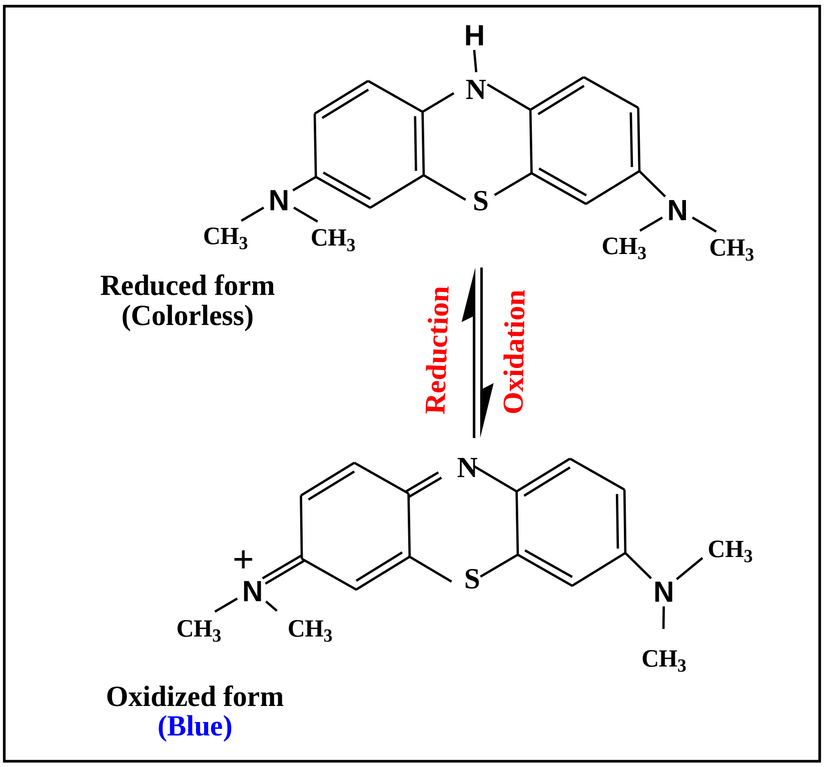

Figure S11. Oxidized and reduced form of methylene blue

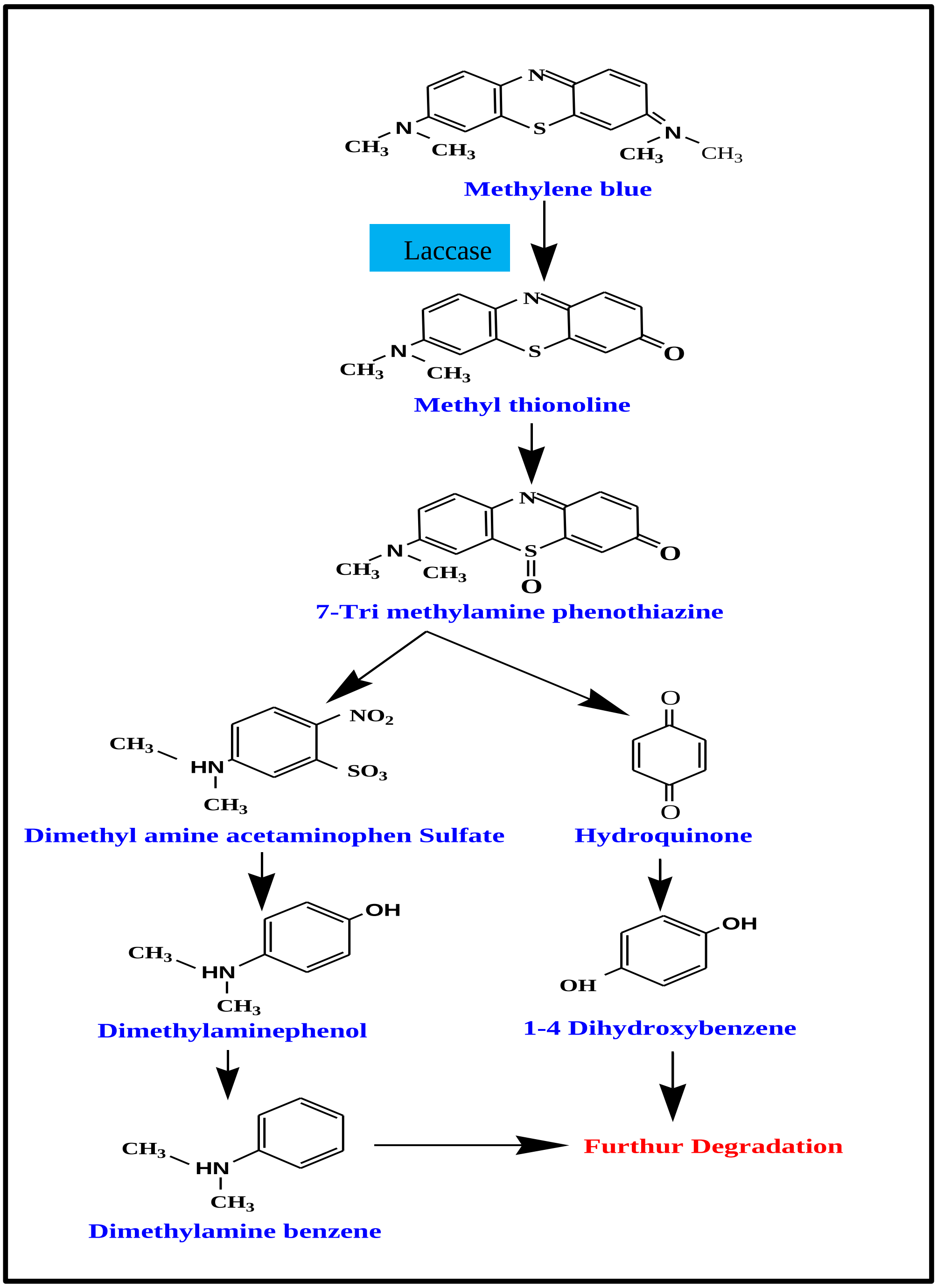

Figure S12. Degradation of methylene blue dye by laccase

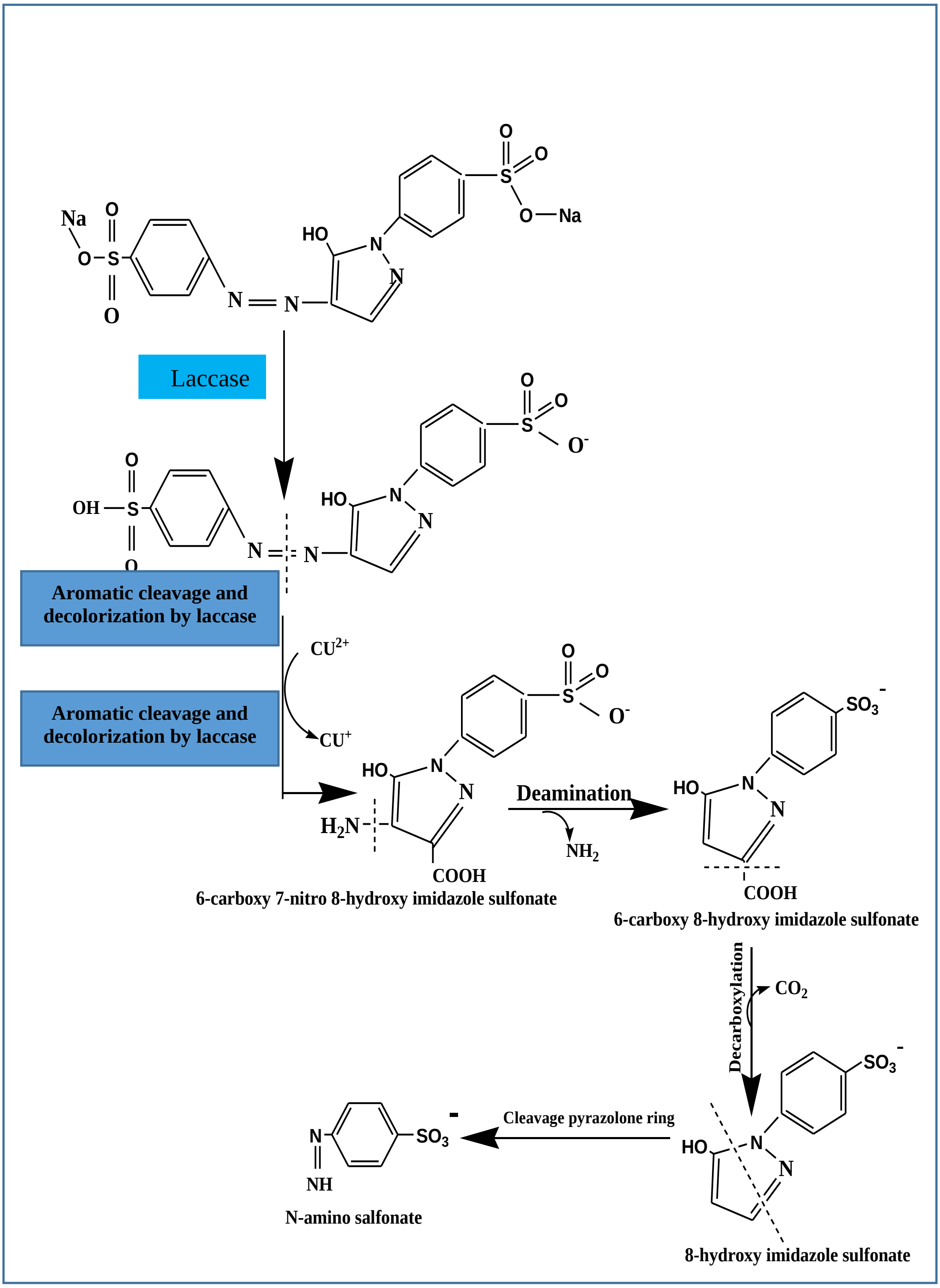

Figure S13: Degradation of thiazine azo dye by laccase

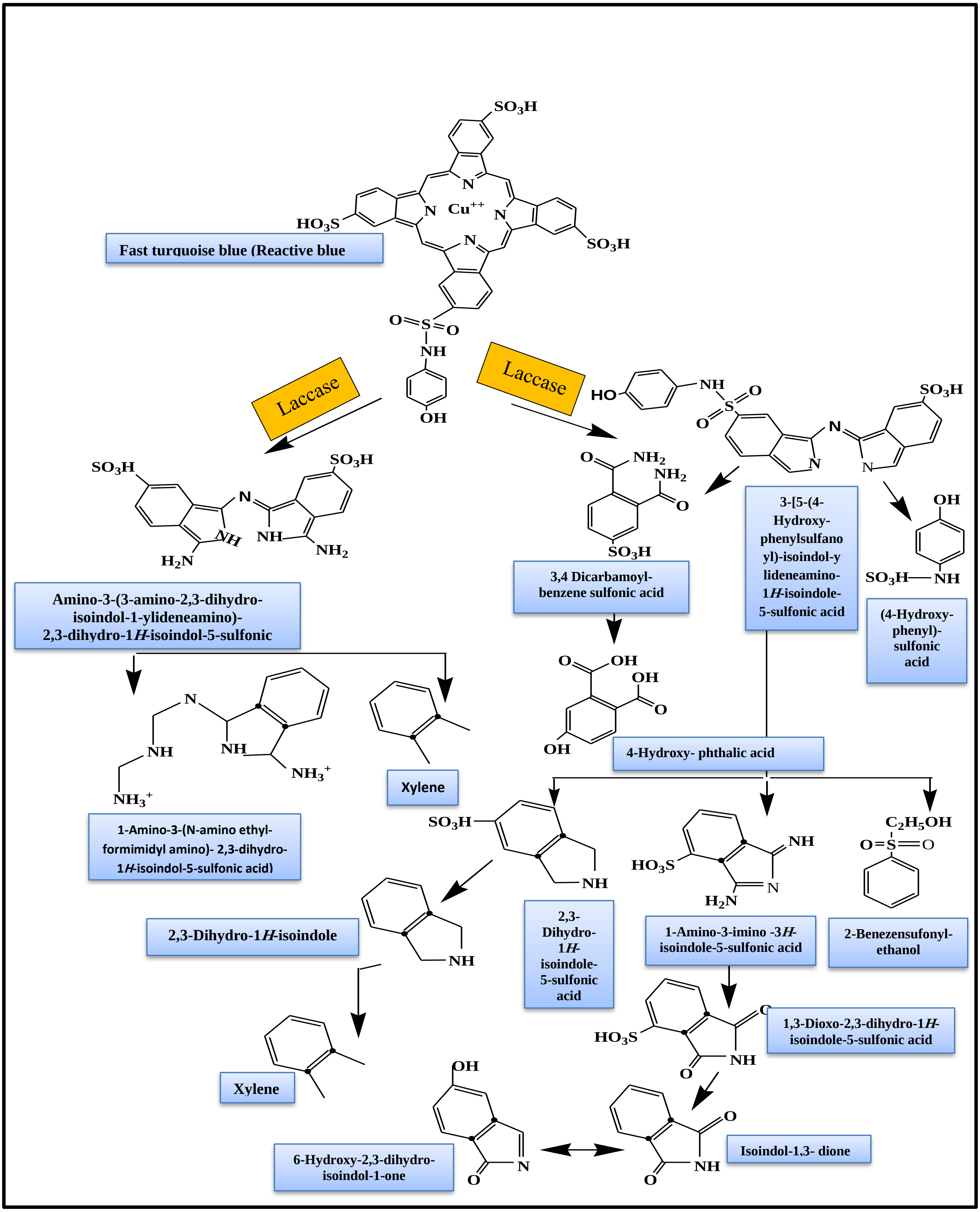

Figure S15. Degradation pathway of fast turquoise blue dye by laccase
